# Facile Synthesis of Nanolayered Manganese Oxide for the Efficient and Selective Removal of Strontium(II) from Nuclear Wastewater

**DOI:** 10.1002/advs.202417776

**Published:** 2025-06-19

**Authors:** Fan Wang, Qi Zheng, Wenya Tai, Qiang Wu, Xinglei Li, Yehuizi Wu, Ningchao Zheng, Shunyan Ning, Deqian Zeng, Hiroshi Watabe, Yan Wu, Hai Li, Yuezhou Wei, Xiangbiao Yin

**Affiliations:** ^1^ School of Nuclear Science and Technology University of South China 28 Changsheng West Road Hengyang 421001 China; ^2^ Institute of Zhejiang University Quzhou 99 Zheda Road Quzhou 324000 China; ^3^ Division of Radiation Protection and Safety Control, Cyclotron and Radioisotope Center Tohoku University 6‐3 Aoba, Aramaki Aoba‐ku, Sendai Miyagi 980‐8578 Japan; ^4^ School of Nuclear Science and Engineering, Shanghai Jiao Tong University 800 Dong Chuan Road Shanghai 200240 China; ^5^ Hangzhou Xiangting Technology Co., Ltd. 1378 Wenyi West Road Hangzhou 314000 China; ^6^ Key Laboratory of Advanced Nuclear Energy Design and Safety Ministry of Education, University of South China 28 Changsheng West Road Hengyang China

**Keywords:** facile synthesis method, nanolayered manganese oxide, nuclear wastewater, strontium‐90 capture

## Abstract

Efficient and selective removal of radioactive ^90^Strontium (^90^Sr) from nuclear wastewater is a sustainable strategy for nuclear energy development and environmental protection due to its long half‐life and high biochemical toxicity. Herein, a novel nanolayered manganese oxide material (Na_0.7_MnO_2.05_, Na‐NLMO) is proposed and its application for Sr(II) capture in both simulated waste liquid and real natural water systems. Na‐NLMO is tailored for Sr(II) adsorption through a simple and rapid solid‐phase reaction, which has good chemical stability and a strong affinity for Sr(II) across the pH range of 1 to 8. The maximum adsorption capacity is 145.21 mg g^−1^ taking advantage of the solid‐liquid ratio of 0.67 g L^−1^. The loaded Sr(II) is almost quantitatively recovered by dilute hydrochloric acid solution. Even after 4 adsorption‐desorption cycles, it could still effectively adsorb more than 99.9% of Sr(II). Furthermore, the adsorption is unaffected by the excess presence of sodium(I), potassium(I), magnesium(II), and calcium(II). In real‐world media such as tap water and river water, its distribution coefficients are 5.2 × 10^5^ and 5.6 × 10^5^ mL g^−1^, respectively. More importantly, the distribution coefficient toward ^90^Sr in seawater still achieved 2.5 × 10^3^ mL g^−1^. The adsorption mechanism of electrostatic interaction and ion exchange is revealed by combining experimental results and spectroscopic analysis, such as Transmission Electron Microscope and X‐ray diffraction. The results are expected to provide new insights into the development of innovative and practical technologies for the treatment and disposal of secondary nuclear waste.

## Graphical abstract

A novel nanolayered manganese oxide material (Na‐NLMO) is prepared using a rapid and straightforward method for efficient adsorption of Sr(II) in both simulated waste liquid and natural water systems. Its excellent adsorption performance of Na‐NLMO could be attributed to the accessible layered structure and negatively charged framework.

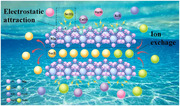

## 1 Introduction

The imperative to address radioactive contamination has been accentuated by the growing demand for sustainable development in nuclear energy, particularly in view of the Fukushima nuclear accident and subsequent discharge of nuclear wastewater into the ocean.^[^ ^1^ ^]^ After the generation of nuclear energy, occurrences of nuclear accidents, and experiments involving nuclear weapons, the produced strontium‐90 (^90^Sr) is considered as one of the most widely distributed radioactive nuclides in the environment and the major contributor to the radiotoxicity of high‐level liquid waste (HLLW).^[^ ^2^ ^] 90^Sr is a relatively long‐lived β‐emitter (t_1/2_ = 28.74 y) with high fission yield (4.5%) and notable environmental migration and radiotoxicity.^[^ ^3^ ^]^ During the Fukushima nuclear accident, a large amount of ^90^Sr was released into the environment, resulting in its persistent presence at a level of 3.7 Bq mL^−1^ in water tanks.^[^ ^4^ ^]^ Furthermore, the chemical similarity of Sr(II) to Ca(II) makes it high bioaccumulation in organisms and leads to diseases such as leukemia or bone cancer.^[^ ^5^, ^6^ ^]^ On the other hand, ^90^Sr has been widely used in nuclear medicine, nuclear batteries, and radiographic thickness measurements.^[^ ^7^ ^]^ Therefore, the quest for efficient materials tailored for the selective extraction of ^90^Sr is an active topic. Such efforts are not only for radioactive pollution remediation and long‐term management of nuclear waste but also for radioactive isotope recovery.

To extract Sr^2+^ ion from aqueous solutions, various methods including ion exchange,^[^ ^8^, ^9^ ^]^ chemical precipitation and flocculation,^[^ ^10^ ^]^ membrane filtration,^[^ ^11^ ^]^ solvent extraction,^[^ ^12^ ^]^ and phytoremediation^[^ ^13^ ^]^ were employed. The ion exchange has emerged as a promising candidate for controlling radioactive pollution and recovering radionuclides due to its high purification factor, treatment efficiency, simplicity of process, and ease of operation. Over the years, various ion exchange materials have been investigated, including nano‐organic materials,^[^ ^14^ ^]^ carbon‐based materials,^[^ ^15^ ^]^ metal‐organic frameworks (MOFs)^[^ ^16^, ^17^ ^]^ and organic resins.^[^ ^18^ ^]^ However, these materials face some major drawbacks in terms of harsh preparation conditions and high costs.^[^ ^19^ ^]^ In addition, organic materials usually have poor irradiation resistance and thermal stability, and are prone to decomposition, leading to secondary contamination, which is better compensated by inorganic materials.^[^ ^20^ ^]^ However, conventional inorganic materials such as clays,^[^ ^21^ ^]^ titanates,^[^ ^22^, ^23^ ^]^ titanosilicates,^[^ ^24^ ^]^ hydroxyapatite,^[^ ^25^ ^]^ and zeolites,^[^ ^26^ ^]^ are generally confined to a narrow pH range and exhibit limited adsorption capacity and affinity for Sr(II).

Recently, inorganic layered 2D materials have demonstrated good ion‐exchange capabilities for caesium, strontium, actinide, and rare‐earth metal ions.^[^ ^27^ ^]^ Similar to other adsorbents, their adsorption performances are notably reduced under more acidic conditions. For instance, layered sodium vanadosilicates achieve ≈95% (distribution coefficient (*K*_d_) > 4 × 10^4^ mL g^−1^) for Sr^2+^ ions removal across a pH range of 3 to 11 in contrast to only ∼5% capture at pH 2.5.^[^ ^28^ ^]^ Fortunately, the development of layered manganese oxide K_0.5_Mn_2_O_4_‐0.6H_2_O (KMO) has improved the acid resistance of this material, achieving a *K*_d_ value of 2.2 × 10^4^ mL g^−1^ for adsorbed Sr^2+^ ions at pH 2. However, the synthesis process was carried out under strongly alkaline conditions, and the limited interlayer spacing of the resulting material constrained its adsorption capacity.^[^ ^29^ ^]^

In this work, the novel sodium‐type nanolayered manganese oxides (Na‐NLMO) was synthesized by a rapid and straightforward solid‐phase calcination method. The preparation process was tailored for the adsorption of Sr(II) ions by fine‐tuning the template agent, Na/Mn molar ratio, and calcination conditions. Comprehensive characterization of the Na‐NLMO was conducted using X‐ray diffraction (XRD), scanning electron microscopy (SEM), thermogravimetric analysis (TGA) and nitrogen adsorption‐desorption measurements. The adsorption kinetics, isotherms and affinity of Na‐NLMO for Sr^2+^ were evaluated and validated using real environmental water samples. The underlying adsorption mechanisms and chemical stability were elucidated through a combination of SEM‐energy dispersive X‐ray spectroscopy (SEM‐EDS), transmission electron microscopy (TEM), Fourier transform infrared spectroscopy (FT‐IR), XRD and X‐ray photoelectron spectroscopy (XPS).

## 2 Results and Discussion

### 2.1 Fast and Facile Synthesis Method

**Figure 1:**
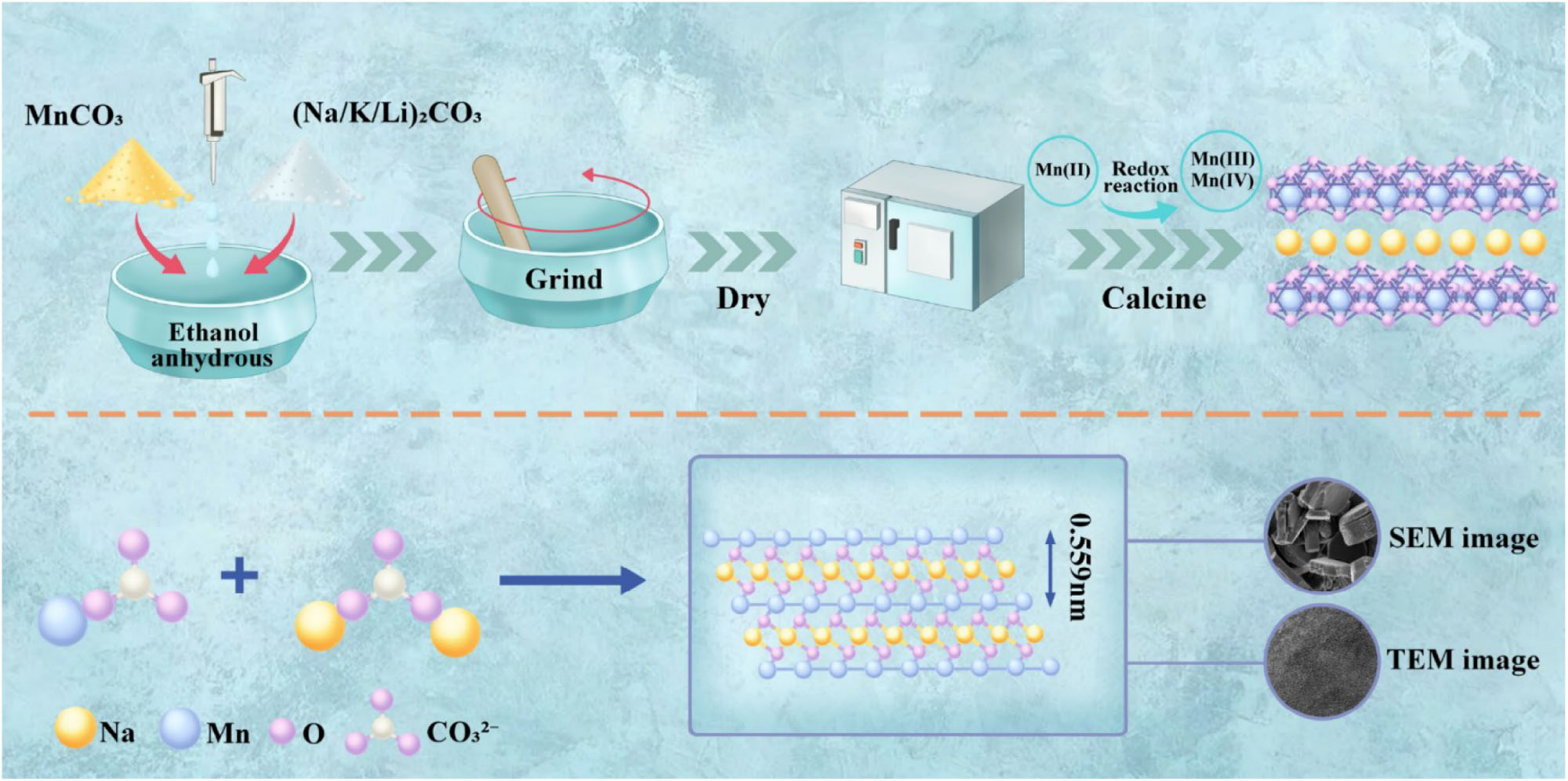
Synthesis schematic of Na‐NLMO.

To the best of our knowledge, solvothermal synthesis was widely recognized as one of the most predominant methods for the preparation of functional materials such as MOFs and polymorphic manganese oxides.^[^ ^30^, ^31^ ^]^ However, this approach usually requires prolonged reaction times and specific conditions, thereby impeding their large‐scale production. Moreover, the preparation of classical Na^+^‐type layered manganese oxides was found to take more than 8 days and involved additional process steps such as pH adjustment.^[^ ^32^ ^]^ To facilitate the development of layered manganese oxide and their application in the separation of strontium, we present a fast and straightforward synthesis method for a novel Na^+^‐type nanolayered manganese oxides (Na‐NLMO). As depicted in **Figure** **1**, a known mass of MnCO_3_ and Na_2_CO_3_, along with a small amount of anhydrous ethanol were placed in an agate mortar at a fixed molar ratio. The mixture was ground for 2 h to obtain a homogeneously dispersed powder. After drying at 80 °C, the powder was calcined in a muffle furnace at 500 °C for 4 h. The resulting solid phase was washed with deionized water and dried again to yield the final strontium ion exchanger Na‐NLMO. The preparation method is simple and time‐efficient, particularly when integrated with corresponding techniques for large‐scale production, it will present greater prospects for this ion exchanger and its application to strontium extraction.

### 2.2 Optimization and Characterization of Na‐NLMO

**Figure 2:**
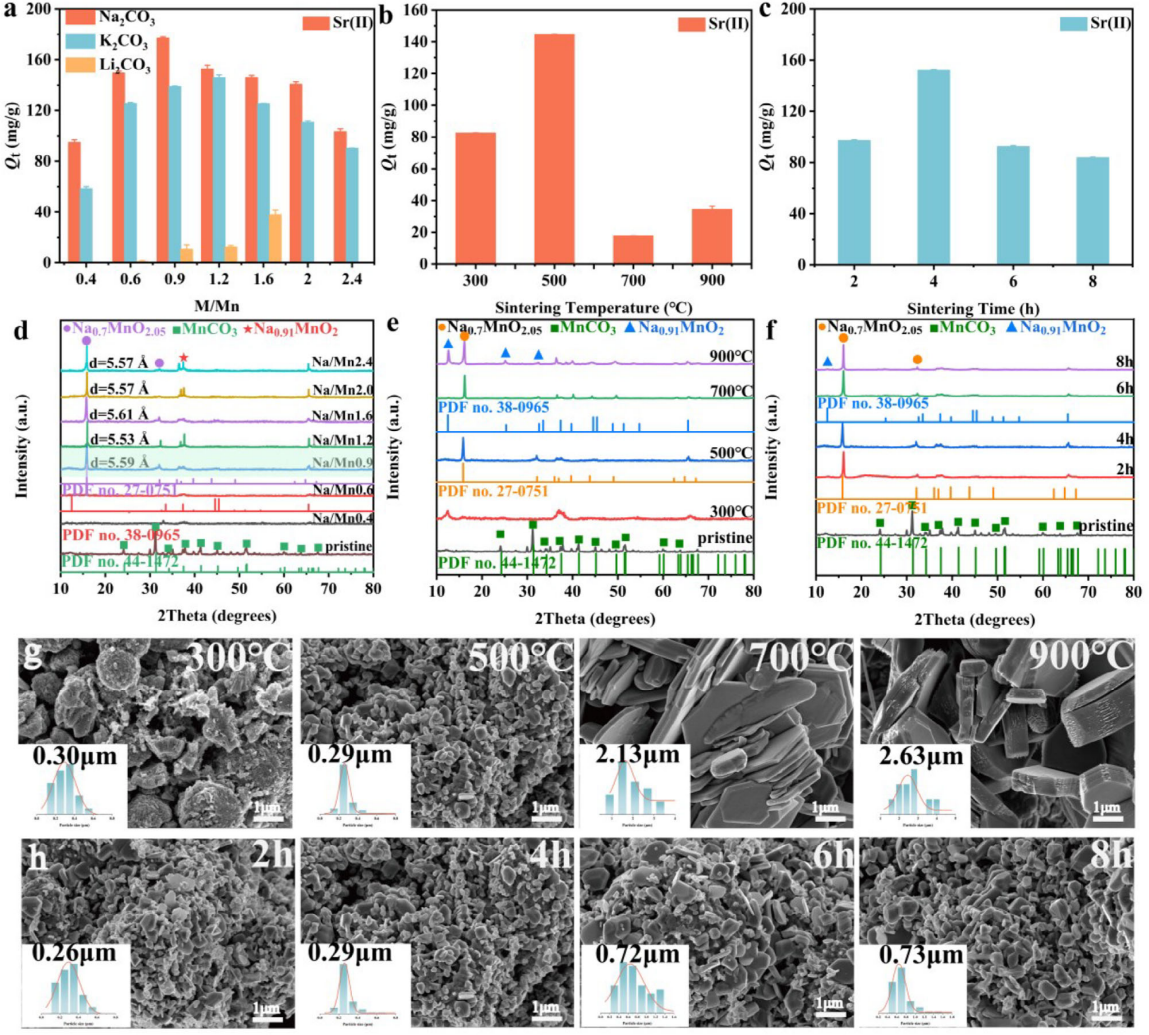
a) Effect of template agent and molar ratio of M/Mn (calcined at 500 °C for 4 h), b) calcination temperature (Na/Mn = 0.9, calcined for 4 h) and c) time (Na/Mn = 0.9, calcined at 500 °C) on the adsorption of Sr(II), [Sr(II)]_initial_ = 500 mg L^−1^, t = 120 min, m/V = 0.67 g L^−1^, pH = 3 at room temperature; (d–f) XRD patterns and g,h) SEM images with of Grain size distribution diagram the Na‐NLMO.

During the preparation of NLMO, one or more alkali metal salts are typically employed as interlayer template agents.^[^ ^33^ ^]^ Considering the similar ionic radii between Li(I), Na(I), K(I) and Sr(II) ions, we initially investigated the impact of doping with sodium carbonate (Na_2_CO_3_), potassium carbonate (K_2_CO_3_), or lithium carbonate (Li_2_CO_3_) on the Sr(II) adsorption by NLMO. As shown in **Figure** **2**a, the adsorption capacity of Sr(II) by Na‐NLMO was significantly higher than that of Li‐NLMO and K‐NLMO. This could be attributed to the closer radius between Na(I) ions (1.02 Å) and Sr(II) ions (1.18 Å).^[^ ^33^ ^]^ Consequently, Na_2_CO_3_ was chosen as the template agents for the NLMO preparation.

The adsorption properties and preparation process of Na‐NLMO were further optimized by changing the doping amount of Na(I). With the increase of the molar ratio of Na/Mn, the material showed an increasing and then decreasing trend in its adsorption capacity for Sr(II), and the maximum value was attained at a molar ratio of 0.9 (Figure 2a). This indicates that over‐doping of Na ions is unfavorable for the ion exchange process. Furthermore, XRD patterns (Figure 2d) reveal the presence of a typical layered structure and the crystal phase of Na_0.7_MnO_2.05_ (PDF No. 27–0751) at diffraction angles of 15.8° and 32.1° when the molar ratio of Na/Mn ranged from 0.9 to 2.4,^[^ ^34^ ^]^ and the corresponding layer spacing was varied from 5.53 to 5.61 Å. It is worth noting that a tunneling structure was observed at 37.4° for molar ratios of Na/Mn of 1.2, 2.0, and 2.4.^[^ ^35^ ^]^ In general, ion exchangers with tunneling structures demonstrate limited cation storage capacity and are prone to ion detachment issues, thereby restricting their exchange efficiency.^[^ ^36^ ^]^ This finding aligns with the adsorption performance of the respective Na‐NLMO for Sr(II).

**Figure 3:**
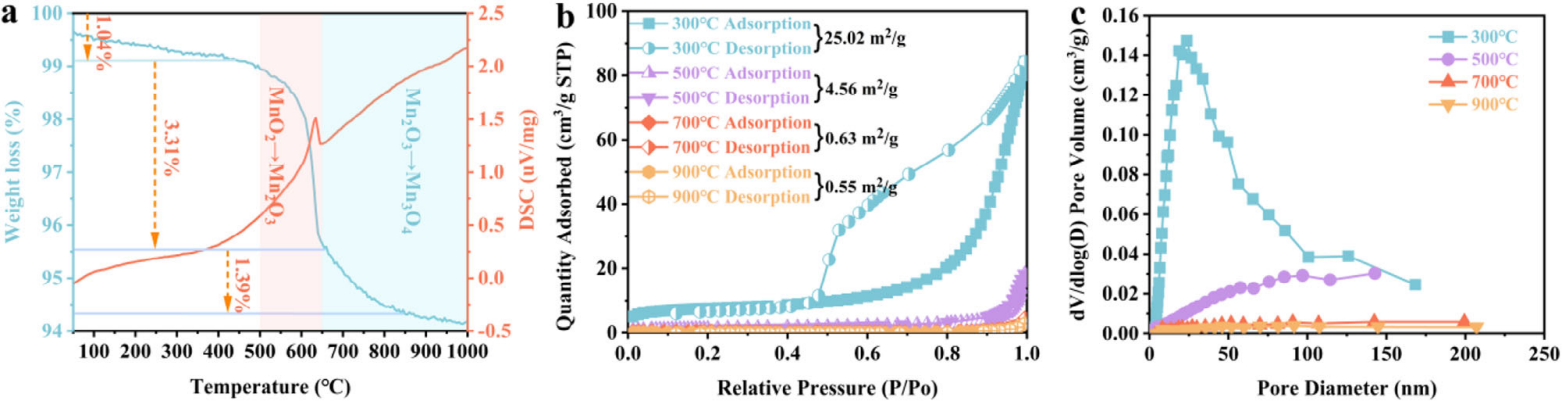
a) TG‐DSC curves, b) N_2_ adsorption‐desorption isotherms, and c) pore size distribution of the corresponding Na‐NLMO samples.

Based on the above studies, the effects of different calcination temperatures and times on the preparation of Na‐NLMO were investigated. When the calcination temperature exceeded 300 °C (Figure 2e), distinct crystalline phases associated with Na_0.7_MnO_2.05_ were consistently observed in the correlating XRD patterns, indicating that lower calcination temperatures (≤ 300 °C) are not favorable for the construction of the desired layered structure. However, the tunneling structure were observed at 12.5°, 25.3°, and 32.6° when the temperatures greater than 500 °C, suggesting the formation of a new crystalline phase, Na_0.91_MnO_2_ (PDF No. 38–0965). SEM images (Figure 2g) showed an increase in the degree of crystallization and size of Na‐NLMO with increasing calcination temperature. The average sizes of Na‐NLMO at 500, 700, and 900 °C were determined as 0.29, 2.13, and 2.63 µm respectively using nano software calculations. As we know, the increase in particle size results in an amplified diffusion distance for target ions, potentially making the exchange process more difficult. According to the characterization of N_2_ adsorption‐desorption isotherms and pore size distributions, the specific surface area of Na‐NLMO gradually decreases with the increase of calcination temperature, and its obvious porous features observed at 300 °C were gradually disappearing. (**Figure** **3**b). Combined with XRD and SEM analyses, this observation could be attributed to the enhanced crystallinity of Na‐NLMO. Therefore, the use of lower or higher calcination temperatures during the preparation process may not be conducive to efficient strontium capture. As expected, the adsorption capacity of Sr(II) by Na‐NLMO synthesized at various calcination temperatures also supported the above results, especially the best adsorption efficiency was obtained at 500 °C (Figure 2b). Furthermore, the formation of crystalline phase of Na‐NLMO was also affected by the calcination time. By integrating the results obtained from XRD and SEM analyses, along with the adsorption properties (Figures 2c,f,h), it was suggested that the optimal crystalline phase for Sr(II) adsorption was achieved after the duration of 4 h. To achieve the optimum removal of Sr(II), the ion exchanger Na‐NLMO was prepared by a moderate temperature solid‐state reaction at 500 °C for 4 h, using a Na/Mn molar ratio of 0.9.

The TG curve of Na‐NLMO was presented in Figure 3a. As seen, the material showed a trend toward thermal decomposition, with a gradual loss of 1.04% of the guest water molecules within the range of 50 and 500 °C. As the temperature increased to 650 °C, a rapid weight loss trend was observed, mainly due to the release of O_2_ from MnO_2_ and the generation of Mn_2_O_3_ by the heat.^[^ ^37^ ^]^ Furthermore, a subsequent weight loss of 1.39% was noted between 650 and 900 °C, which could be ascribed to extended thermal exposure resulting in the formation of Mn_3_O_4_ and simultaneous release of O_2_.^[^ ^37^ ^]^ In general, even when subjected to the heating condition of 900 °C, the present Na‐NLMO experienced only a 5.74% weight loss, indicating exceptional thermal stability. Interestingly, the entire process was accompanied by the disappearance of Mn^4+^ ions and the generation of Mn^3+^ ions, which primarily located in the vacancies on the MnO_6_ octahedra.^[^ ^32^ ^]^ This transformation facilitated the outward migration of Na^+^ ions between the layers to uphold charge equilibrium. Consequently, the reduction in the interlayer Na^+^ content results in a decreased ion exchange capacity for the material. These observations were consistent with the findings in Figure 2 (b,e,g).

### 2.3 Adsorption Performance

To evaluate the adsorption ability of Na‐NLMO toward Sr(II) and its potential for environmental remediation, the effect of acidity on Sr(II) adsorption, as well as adsorption kinetics and isotherms were investigated.

#### 2.3.1 Effect of Acidity

**Figure 4:**
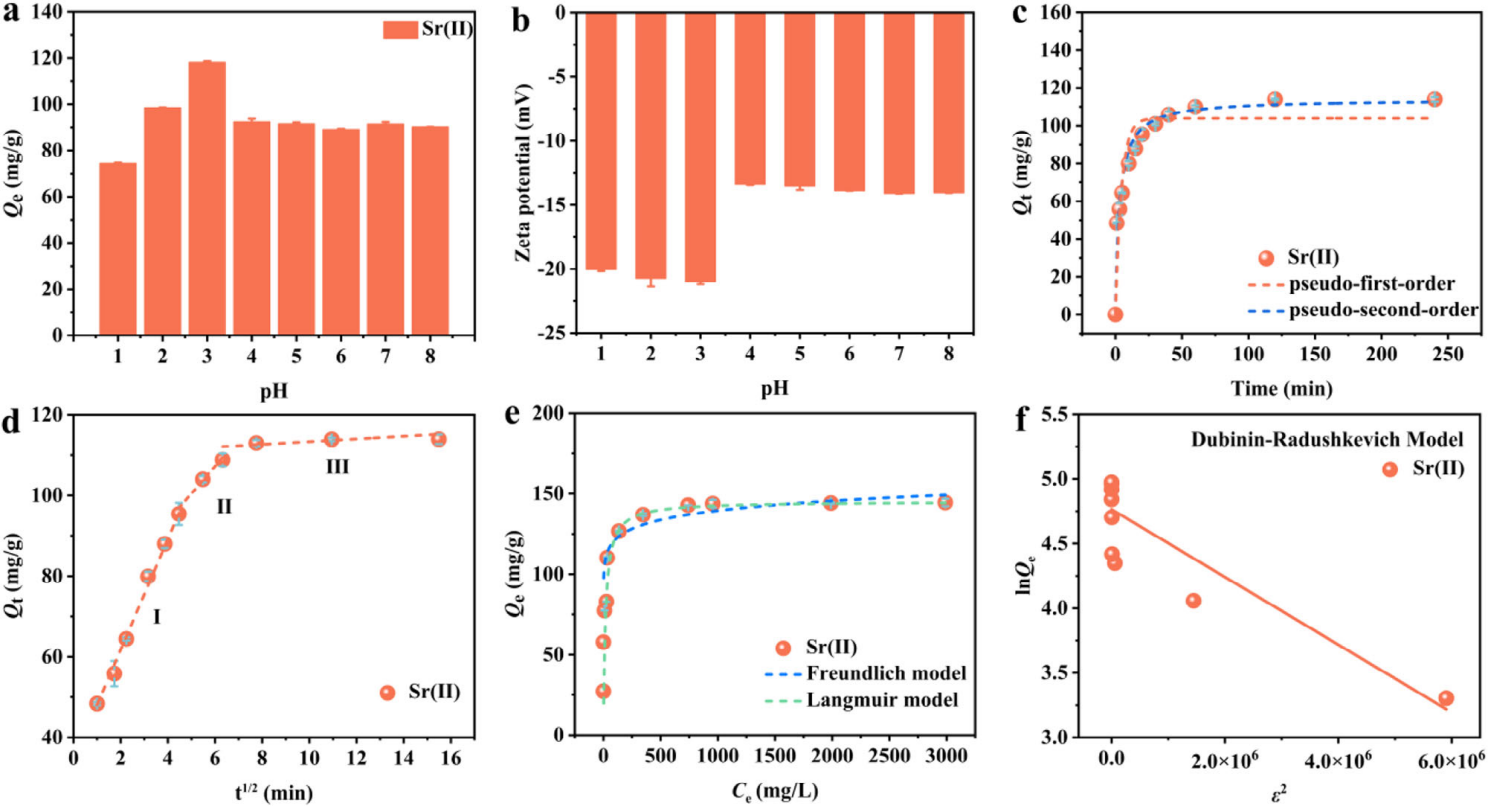
a) Effect of pH on the adsorption capacity of Na‐NLMO for Sr^2+^; [Sr(II)]_initial_ = 200 mg L^−1^, t = 120 min, m/V = 0.67 g L^−1^ at room temperature; b) Zeta potential value of Na‐NLMO with different pH; c) Effect of contact time on Sr(II) adsorption and the kinetic behavior fitted using pseudo‐first‐order, pseudo‐second‐order and d) intraparticle diffusion models; Adsorption isotherms of Sr(II) by Na‐NLMO and fitting results using e) Langmuir, Freundlich and f) D‐R models.

**Figure** **4**a illustrates the effect of acidity on the adsorption of Sr(II) by Na‐NLMO. It is evident that the adsorption capacity increase and then decrease as the pH value rises, the maximum adsorption capacity of 118.16 mg g^−1^ was obtained at pH 3, and ultimately remains almost constant at pH values between 4 and 8. As demonstrated by the zeta potential analysis (Figure 4b), the adsorption behavior of Na‐NLMO was clearly influenced by the surface potential. At pH 3, a more negative potential were observed, indicating that the affinity of Na‐NLMO for Sr(II) ions was optimal under this condition. In fact, the hexagonal structure of manganese oxides predominantly displays charge defects attributed to vacancies, edge sites, and low‐valence manganese ions.^[^ ^38^ ^]^ Consequently, at pH levels above 3, H^+^ ions from the solution could infiltrate the edge sites of the adsorbent, effectively neutralizing these charge defects.^[^ ^39^ ^]^ This neutralization process ensures an optimal concentration of Na⁺ ions within the interlayers, thereby enhancing the efficiency of ion exchange. However, as the acidity increases, the excess H^+^ ions could compete with the target cations, leading to the reduction in adsorption efficiency.

#### 2.3.2 Adsorption kinetics

To determine the kinetic parameters of Sr(II) adsorption by Na‐NLMO, the influence of contact time on adsorption was examined. Na‐NLMO offered faster capture of Sr(II), the adsorption capacity increased to 64.45 mg g^−1^ within the first 5 min (Figure 4c). After the contact of 120 min, the ion exchange process reached an equilibrium state with the adsorption capacity of 113.91 mg g^−1^. Based on the above results, the pseudo‐first‐order^[^ ^40^ ^]^ and pseudo‐second‐order kinetic models^[^ ^41^ ^]^ were employed to assess the kinetic behavior of Na‐NLMO. In addition, the intraparticle diffusion model^[^ ^42^ ^]^ was utilized to investigate the adsorption mechanism and rate‐controlling steps. As shown in Figure 4c and **Table** **1**, the adsorption process of Sr(II) by Na‐NLMO was more suitably described by the pseudo‐second‐order model (R^2^ = 0.96), and the equilibrium adsorption capacity was almost the same as the theoretical value (*Q*_e, cal_ = 114.21 mg g^−1^), which suggested that the adsorption process was dominated by the chemical interactions. Figure 4d and Table 1 depicts the results fitted by the intra‐particle diffusion model, indicating the adsorption process of Sr(II) by Na‐NLMO can be divided into three distinct phases. Furthermore, the absence of curve intersections at the origin implied that internal diffusion is not the sole rate‐limiting step in the adsorption process.

**Table 1:** Kinetics parameters for the adsorption of Sr(II) by Na‐NLMO.

model	Parameter	
	*Q*_e,exp_ (mg/g)	113.91
pseudo‐first‐order	*Q*_e,cal_ (mg/g)	103.89
	*k*_1_(×10^−4^)	2214.31
	*R* ^2^	0.88
pseudo‐second‐order	*Q*_e,cal_ (mg/g)	114.21
	*k*_2_(×10^−4^)	2.80
	*R* ^2^	0.96
intraparticle diffusion	*k*_id_ (mg/(g min^1/2^))	13.69
	C	34.41
	*R* ^2^	0.996

#### 2.3.3 Adsorption Isotherms

The adsorption isothermal behavior of Na‐NLMO was investigated in Sr(II) solutions ranging from 20 to 3000 mg L^−1^. As shown in Figure 4e, the adsorption capacity gradually increased with the increase of Sr(II) concentration, and tends to stabilize after reaching the maximum capacity (144.52 mg g^−1^). Subsequently, the Langmuir,^[^ ^43^ ^]^ Freundlich,^[^ ^44^ ^]^ and Dubinin‐Radushkevich (D‐R) models^[^ ^45^ ^]^ were employed. The fitting results (**Table** **2**) indicate that the Langmuir model is more suitable than the Freundlich model, suggesting that the adsorption process of Na‐NLMO aligns with the monolayer adsorption mechanism. Meanwhile, the maximum adsorption capacity of Na‐NLMO for Sr(II) was determined to be 144.52 mg g^−1^, which closely approximates the theoretical value (145.21 mg g^−1^). More importantly, the higher adsorption capacity at a lower solid‐liquid ratio of 0.67 g L^−1^ not only minimizes the secondary waste from the used adsorbent but also substantially reduces overall process costs. In the case of the D‐R model (Figure 4f; Table 2), the free energy of Sr(II) was 1.39 kJ mol^−1^ (< 8 kJ mol^−1^), which revealed that the adsorption process also involves physisorption. Due to the presence of charge defects introduced by low‐valent manganese ions in the hexagonal structure of manganese oxides, the physical adsorption for Sr(II) ions was triggered by electrostatic interaction. This finding was further supported by the observation of negative zeta potential (Figure 4b).

**Table 2:** Adsorption isotherm parameters of Sr(II) adsorption by Na‐NLMO.

Isotherm model	Parameter	
	*Q*_e,exp_ (mg/g)	144.52
Langumuir	*Q*_max_ (mg/g)	145.21
	*K*_L_	0.05
	*R* ^2^	0.94
Freundlich	*K*_F_ (mg/g)	91.44
	n	16.67
	*R* ^2^	0.91
Dubinin‐Radushkevich	*Q*_max_ (mg/g)	117.16
	*β*	2.6×10^−7^
	*E*	1.39
	*R* ^2^	0.76

### 2.4 Adsorption Mechanism

**Figure 5:**
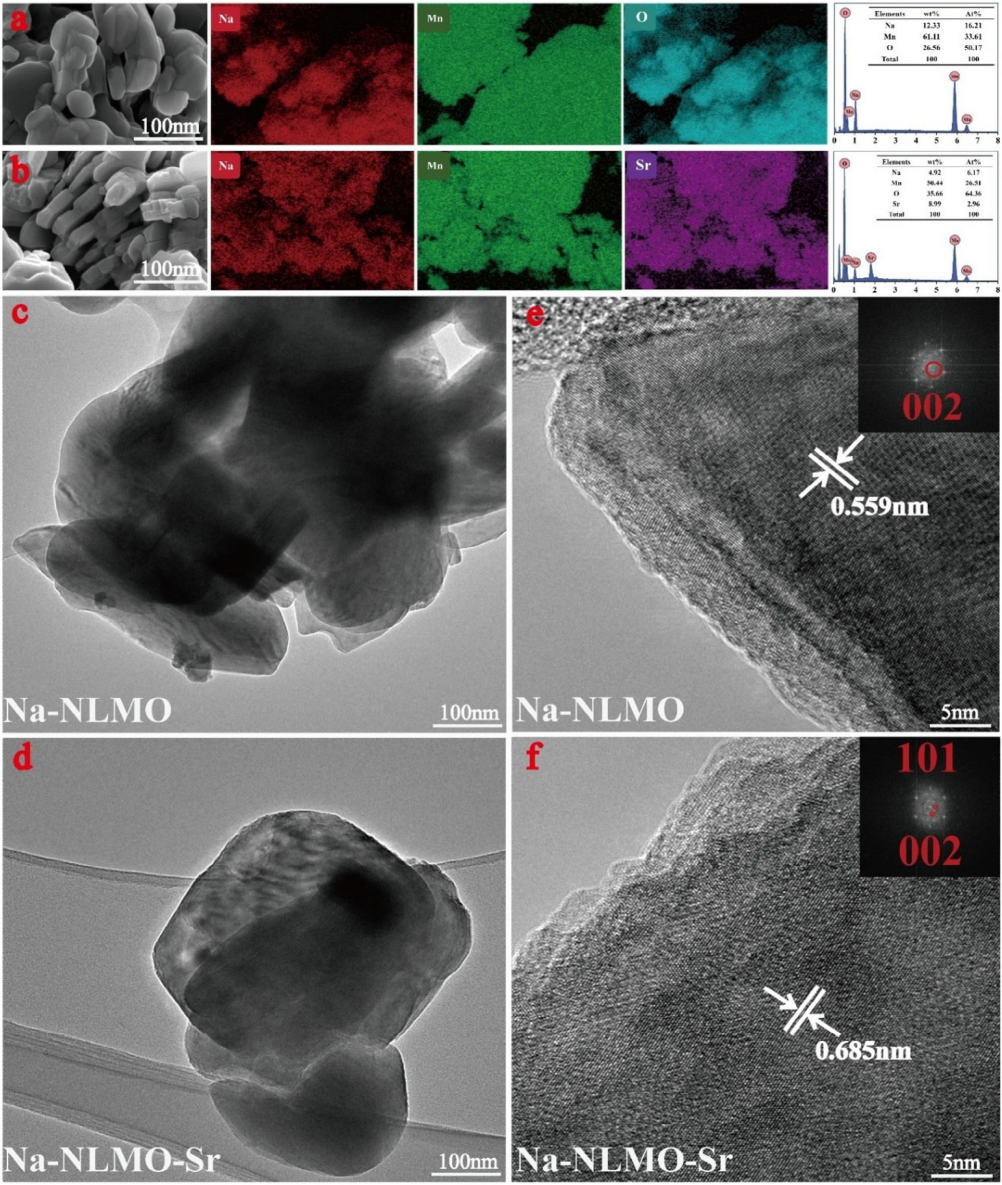
a,b) SEM‐EDS before and after Sr(II) adsorption, c,d) TEM and e,f) High resolution TEM images of the Na‐NLMO c,e) before and d,f) after Sr(II) adsorption.

To gain new insight into the mechanism in terms of Sr(II) adsorption by Na‐NLMO, the characterizations of Na‐NLMO before and after adsorption were performed using SEM‐EDS, TEM, FT‐IR, XRD, and XPS. As illustrated in the SEM and TEM images (**Figure** **5**a–f), the morphology of the Sr(II)‐loaded Na‐NLMO (Na‐NLMO‐Sr) remained unchanged, and the particle sizes were all in the hundreds of nanometers, indicating their high structural stability. The corresponding EDS spectra revealed the uniform distribution of Sr(II) on the surface of material, suggesting that the Sr(II) ion was successfully loaded. It is noteworthy that the content of elemental Na was decreased from 12.33 wt% to 4.92 wt%, which provided evidence for ion exchange during the adsorption process. In addition, the lattice fringes of pristine Na‐NLMO were distinctly visible in the TEM image (Figure 5e), and the inter‐fringe distance was ≈0.559 nm. This measurement aligns with the interlayer spacing obtained in the results of the XRD analysis. After the adsorption, the distance expanded to 0.685 nm, thus further confirming that Sr(II) ions were exchanged into the interlayer of Na‐NLMO.

**Figure 6:**
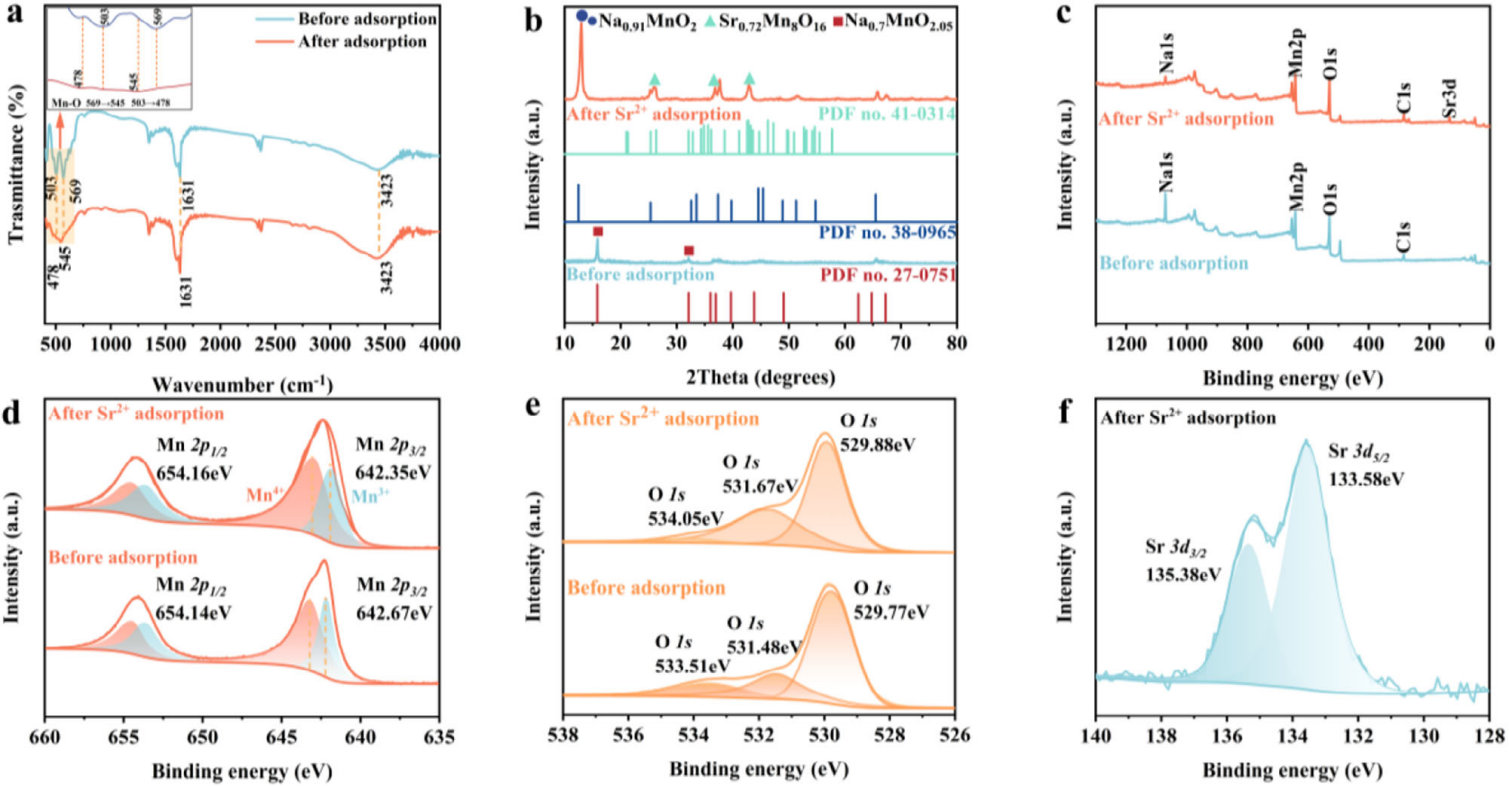
a) FTIR spectra, b) XRD patterns, c) XPS survey, high resolution XPS spectra of d) Mn *2p* and e) O *1s* of the Na‐NLMO before and after Sr(II) adsorption; f) high resolution XPS spectra of Sr *3d* of the Na‐NLMO after adsorption.

**Figure** **6**a shows the FT‐IR spectra of Na‐NLMO before and after strontium adsorption, where the peaks at 3423 cm^−1^ and 1631 cm^−1^ were attributed to the stretching and bending vibrations of the hydroxyl groups in the adsorbed water on the Na‐NLMO.^[^ ^46^ ^]^ The peaks at 503 cm^−1^ and 569 cm^−1^ correspond to the O─Mn─O stretching vibrations within the MnO_6_ octahedron.^[^ ^47^, ^48^ ^]^ For Na‐NLMO‐Sr, the peak was obviously weakened and blue‐shifted to 478 cm^−1^ and 545 cm^−1^, which confirms the successful exchange of Sr(II) for Na(I) and highlights the interaction between Sr(II) and Mn─O─ group. The XRD patterns (Figure 6b) revealed additional diffraction peaks after Sr(II) adsorption, corresponding to Sr_0.72_Mn_8_O_16_ (PDF No. 41–0314), demonstrating the effective adsorption of Sr(II) by Na‐NLMO.

XPS analysis was performed to further explore the interaction of strontium with Na‐NLMO. Compared with the XPS survey of Na‐NLMO before and after adsorption (Figure 6c), the introduction of Sr(II) ions resulted in a new peak corresponding to the characteristic Sr 3d peak, along with the significant decrease in the intensity of the Na 1s peak. Furthermore, the Sr 3d spectrum can be resolved into two subpeaks located at 133.58 and 135.38 eV (Figure 6f), corresponding to Sr 3d5/2 and Sr 3d3/2, respectively.^[^ ^49^ ^]^ These findings suggest that an ion exchange process takes place between Sr(II) and Na(I). For the high‐resolution XPS spectra of Mn 2p (Figure 6d), the coexistence of Mn(III) and Mn(IV) in Na‐NLMO was clearly confirmed, consistent with the findings from the TG analysis. As shown in Figure 6e, the high‐resolution O 1s XPS spectrum of the Na‐NLMO was deconvoluted into three distinct peaks at 529.77, 531.48 eV, and 533.51 eV, corresponding to lattice oxygen, surface oxygen, and adsorbed molecular water, respectively.^[^ ^50^ ^]^ Combined with XRD analysis, the crystalline phase of Na_0.7_MnO_2.05_ gradually transformed into Sr_0.72_Mn_8_O_16_ following ion exchange. As a result, the three peaks in the O 1s XPS spectrum of the adsorbed material were modified.

### 2.5 Reusability

**Figure 7:**
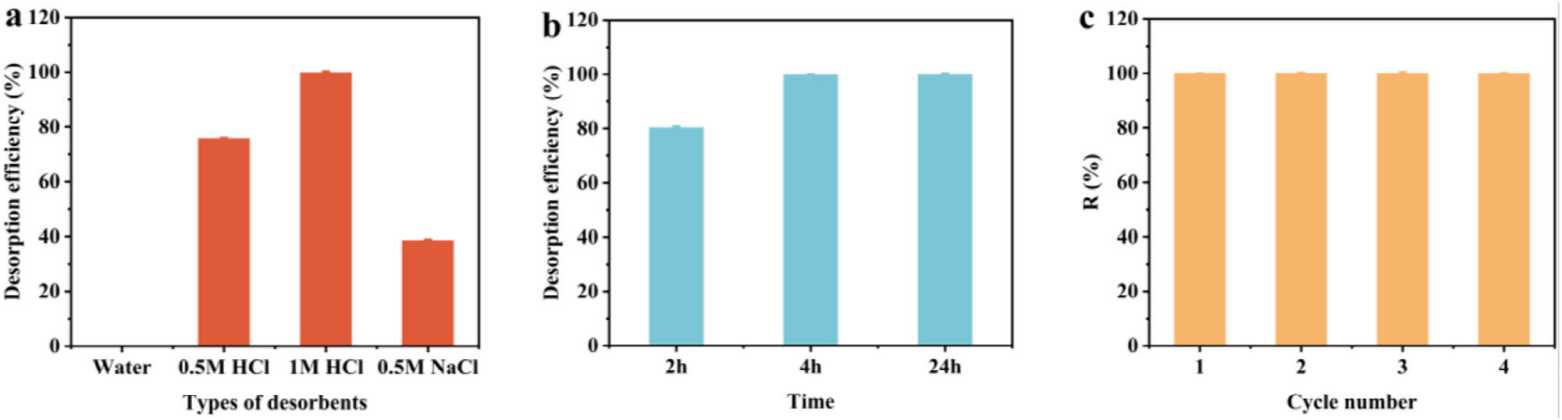
Effect of a) eluent and b) contact time on desorption efficiency; c) The adsorption efficiency of Na‐NLMO toward Sr(II) under four adsorption‐desorption cycles. [Sr(II)]_initial_ = 5 mg Lm, m/V = 0.67 g Lm at room temperature.

To ensure the effective recovery and regeneration of the adsorbent, it is crucial to investigate the elution behavior after adsorption. This step is essential for achieving cost‐effective and scalable industrial application of Na‐NLMO. To this end, deionized water, dilute hydrochloric acid, and NaCl solutions were chosen as desorbents to assess their regeneration capabilities for Na‐NLMO‐Sr. Similar to the adsorption experiments, the solid‐liquid ratio of 0.67 g L^−1^ was also employed for desorption. As shown in **Figure** **7**a, the desorption efficiency of deionized water was almost negligible. Furthermore, it was evident that the desorption efficiency of the 0.5 m HCl solution was higher than that of the 0.5 m NaCl solution. Compared to 0.5 m NaCl (Figure S4, Supporting Information), the desorption efficiency did not increase with increasing concentration of NaCl. This is primarily attributed to the affinity of the present material for Sr(II) is much higher than that of Na(I). In the case of EDTA‐2Na, the desorption efficiency remained below 45%. Notably, the desorption efficiency was afforded to more than 99.7% when the HCl concentration was increased to 1.0 m. Therefore, dilute hydrochloric acid was selected as the desorbent to conduct further adsorption‐desorption cycle experiments. In combination with the effect of contact time (Figure 7b), the loaded Sr(II) was nearly quantitatively enriched by the 1.0 M HCl solution within 4 h. The regenerated material was then utilized in the subsequent adsorption cycle. Even after four adsorption‐desorption cycles (Figure 7c), more than 99.9% of Sr(II) was still effectively adsorbed, highlighting the excellent reusability of the present material. The structural stability of Na‐NLMO was evaluated. As shown in the XRD patterns and SEM images (Figures S5 and S6, Supporting Information), the main structure and morphology of Na‐NLMO were well maintained after four cycles.

### 2.6 Selectivity and Stability

**Figure 8:**
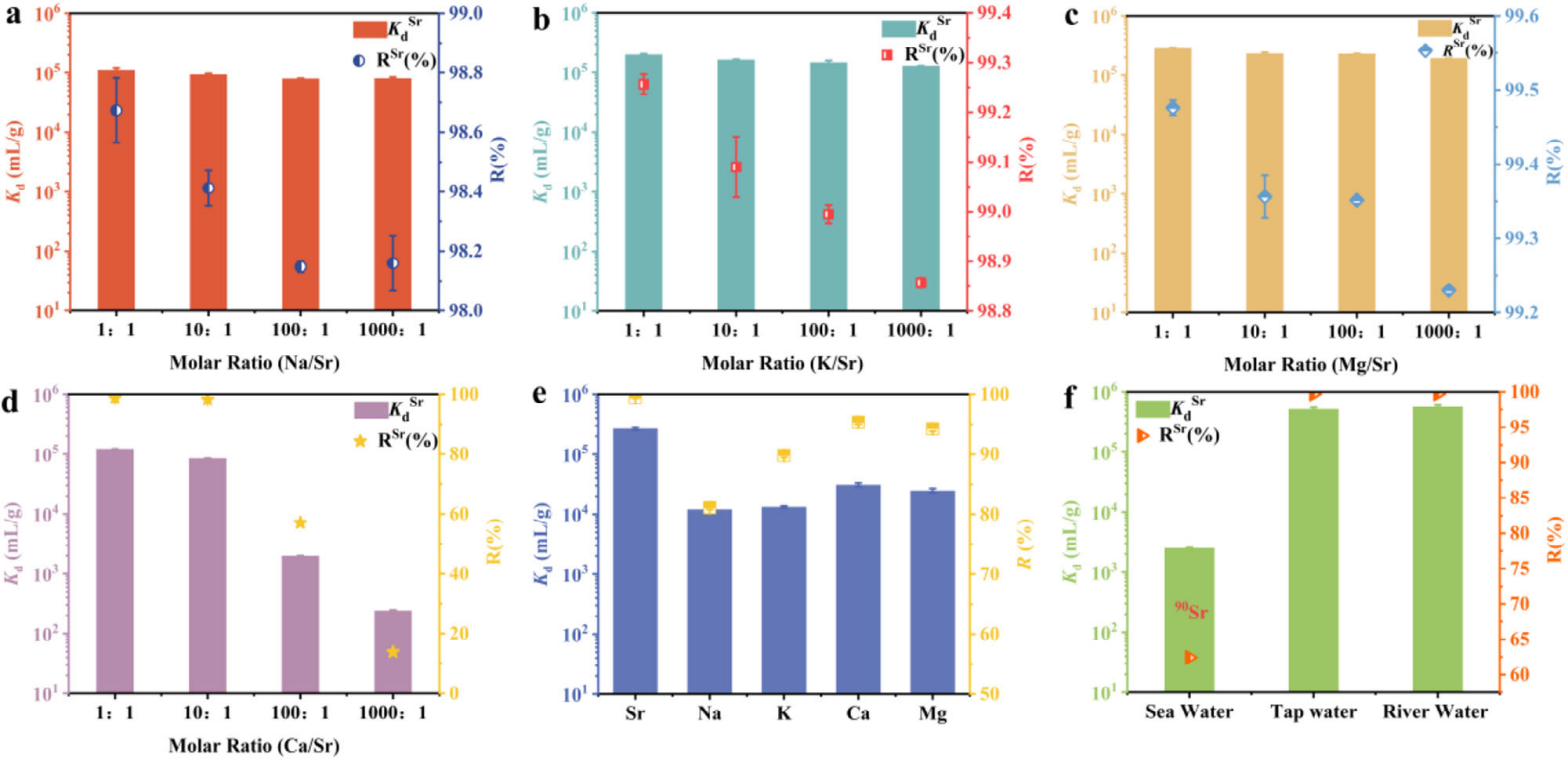
Distribution coefficients (*K*_d_) for Sr(II) removal under different molar ratios of a) Na/Sr, b) K/Sr, c) Mg/Sr, d) Ca/Sr; [Sr(II)]_initial_ = 5 mg L^−1^, pH = 3; e) *K*_d_ for Sr(II) removal in mixed system, [Sr(II)] = [Na(I)] = [K(I)] = [Ca(II)] = [Mg(II)] = 5 mg L^−1^, pH = 3; f) *K*_d_ for Sr(II) removal in seawater, tap water and river water. t = 120 min, m/V = 0.67 g L^−1^ at room temperature.

The composition of environmental water samples is highly complex, containing a wide range of elements at elevated concentrations. Specifically, alkali metal ions (such as Na(I) and K(I)) and alkaline earth metal ions (such as Ca(II) and Mg(II)), share similar properties with Sr(II) and could interfere with the extraction of Sr(II) from the material.^[^ ^51^ ^]^ To evaluate the anti‐interference capability of the developed Na‐NLMO, its adsorption efficiency of Sr(II) in the presence of common competitive cations was investigated. The **Figure** **8**a–c demonstrate that Na‐NLMO was still able to capture Sr(II) even when the molar ratios of Na/Sr, K/Sr, and Mg/Sr reached 1000:1, with the *K*_d_ value remaining unaffected at ≈1 × 10^5^ mL g^−1^. In fact, Ca and Sr share a high degree of chemical similarity. While the adsorption towards Sr(II) by Na‐NLMO was kept the original efficiency when the molar ratio of Ca/Sr reached 10:1. Moreover, the ion‐exchange capacity remained robust even when the concentration of Ca(II) was further increased to 100‐fold and 1000‐fold, with *K*_d_ values of 1.99 × 10^3^ and 2.42 × 10^2^, respectively (Figure 8d). As expected, the present Na‐NLMO was endowed with the ability to remove more than 99% of Sr(II) from the aqueous phase in the presence of the above five cations (Figure 8e). These results imply that Na‐NLMO has a specific affinity for Sr(II).

Given the demonstrated superior extraction capability for strontium and unique resistance to interference, the next critical step was to evaluate the practical utility and application potential of Na‐NLMO for Sr(II) separation in real environmental water samples. Natural water samples often contain Na(I), K(I), Ca(II), and Mg(II) at much higher concentrations than Sr(II), presenting a significant challenge. As shown in Figure 8f, Na‐NLMO exhibited a preference for strontium with *K*_d_ values of 5.2 × 10^5^, and 5.6 × 10^5^ mL g^−1^ in real media of tap water and river water, respectively. To simulate contaminated seawater, 30 mL of seawater samples were spiked with a standard solution of ^90^Sr to give a total β‐activity of 6010 cpm. After contacted with 20 mg of Na‐NLMO, the total β‐activity was reduced to 2240 cpm, showing a removal efficiency of ≈63%. The distribution coefficient of Na‐NLMO toward ^90^Sr in seawater was estimated to be 2.5 × 10^3^ mL g^−1^ when the detection efficiency of the liquid scintillation counter for ^90^Sr was 100%.

**Figure 9:**
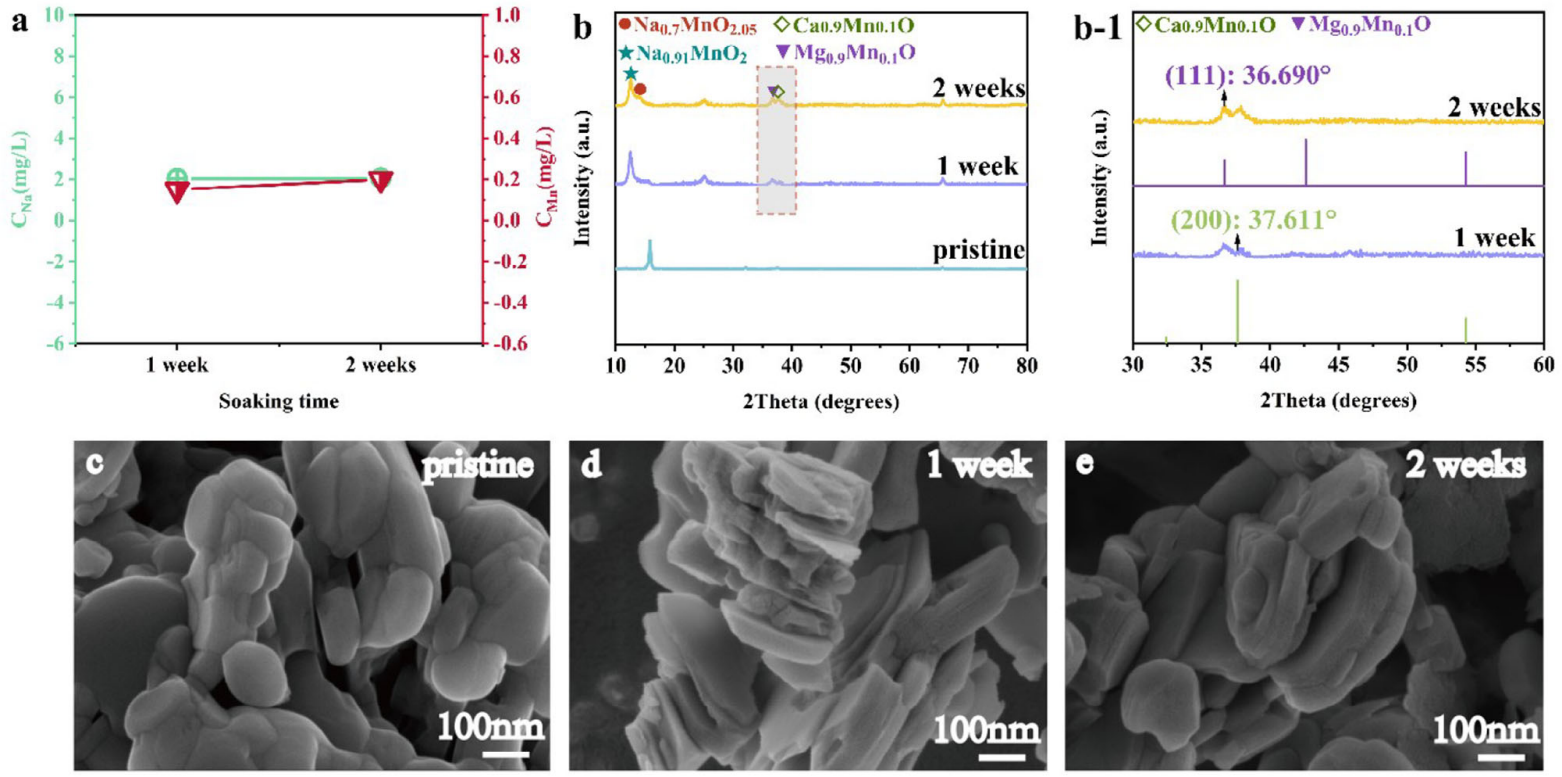
a) Effect of soaking time in seawater on the dissolution behavior of Na and Mn in Na‐NLMO; b) XRD patterns, (b‐1) enlarged patterns and c–e) SEM images of Na‐NLMO after immersion in seawater for different durations.

The stability of Na‐NLMO in seawater was investigated. Only 2 mg L^−1^ of Na ions were leached out after the material was immersed in real seawater for 2 weeks (**Figures** **9**a; S7a, Supporting Information), which accounted for ≈0.2%. Furthermore, manganese ions exhibited negligible dissolution. The XRD pattern (Figures 9b; S7c, Supporting Information) demonstrates that Na‐NLMO retains distinct diffraction peaks corresponding to the layered structure at 15.8° and 32.1°, even after 2 weeks of exposure to seawater. In addition, new crystalline phases of Mg_0.9_Mn_0.1_O (PDF No. 36–1377) and Ca_0.9_Mn_0.1_O (PDF No. 36–1378) were observed during immersion, which could be attributed to the incorporation of Ca(II) and Mg(II) ions from seawater into the material. With prolonged soaking and continued adsorption of these ions, some of the layered structures transform into tunnel structures, thereby enhancing the structural stability of the material and reducing the risk of collapse. This result was also evident in the TEM images. As shown in Figure S9 (Supporting Information), clear lattice fringes persist with time, while the interlayer spacing decreases slightly, which was consistent with the adsorption of Ca(II) and Mg(II). SEM images (Figures 9c–e; S8, Supporting Information) and FT‐IR spectra (Figure S7b, Supporting Information) show that the morphology and the main structure of Na‐NLMO were essentially unchanged after two weeks of immersion. It is obvious that the present Na‐NLMO has good stability and a strong affinity for Sr(II), indicating its potential for removal and recovery of Sr(II) from environmental water systems. Compared with other materials listed in **Table** **3**, the novel Na‐NLMO demonstrated superior separation performance for Sr(II) in terms of adsorption capacity, *K*_d_ value, interference‐resistant, and operational conditions. In particular, Na‐NLMO was facile to synthesize and employed inexpensive raw materials, making it a highly promising candidate for environmental remediation and isotope recovery.

**Table 3:** Comparisons between Na‐NLMO and other reported adsorbents for Sr(II) adsorption.

Adsorbents	*q*_m_ (mg g^−1^)	*K_d_*	Competing ions	pH	Refs.
SZ‐4	118.29	1.32×10^5^	Na^+^, Mg^2+^	2‐8	[7]
FJISM‐SnS	65.19	2.91×10^4^	Na^+^, Mg^2+^, Ca^2+^, Cs^+^	6	[52]
KMS‐2	86.89	2.10×10^4^	Cs^+^, Ni^2+^	7	[53]
TMP‐11	12.27	<10^4^	Na^+^	7	[54]
FJSM‐SnS‐2	59.41	6.58×10^2^	Na^+^, Mg^2+^, Ca^2+^, Cs^+^	3‐12	[55]
AMP‐PAN	16.16	8.66	Na^+^, Co^2+^, Ca^2+^, Cs^+^	>6.5	[56]
K_4_Nb_6_O_17_	155.96	1060	K^+^, Na^+^, Mg^2+^, Ca^2+^	3‐14	[57]
KZP	25.41	3×10^4^	Na^+^, Al^3+^, Ca^2+^	7	[58]
KZTS	19.30	1923	Na^+^, Mg^2+^, Ca^2+^	8	[59]
ZrP	198.44	3500	K^+^, Na^+^, Mg^2+^, Cu^2+^, Ca^2+^	5‐10	[60]
MOF‐18Cr6	84.93	1.14×10^5^	K^+^, Na^+^, Mg^2+^, Cu^2+^, Ca^2+^	4‐10	[16]
CHA	12.41	180	Mg^2+^, Ca^2+^	7	[61]
Sb(III)/Sb_2_O_5_	15.20	10^3^	K^+^, Na^+^, Ca^2+^	2‐8	[62]
Eu‐ox	92.17	2.61×10^5^	K^+^, Na^+^, Mg^2+^, Ca^2+^	4‐10	[10]
WO_3_/SiO_2_	8.73	2000	Mg^2+^, Ca^2+^, La^3+^, Dy^3+^	4‐7	[46]
Na‐NLMO	145.21	5.6× 10^5^	K^+^, Na^+^, Mg^2+^, Ca^2+^	1‐8	This work

## 3 Conclusion

A novel Na‐NLMO was conveniently prepared by the solid‐phase reaction of manganese carbonate and sodium carbonate. The type of templating agent, the molar ratio of Na/Mn, and the calcination temperature and time in the preparation process were specifically decorated for strontium separation based on XRD, SEM, TG‐DSC, BET analyses, and absorption experiments. The combination of the fast and simple synthesis method and cost‐effective precursors demonstrates great promise for large‐scale production and application. Na‐NLMO provides good chemical stability, excellent adsorption capacity (145.21 mg g^−1^), high resistance to cationic interference, quantitative recovery of Sr(II) ions, good regeneration performance, low solid/liquid ratio process, moderate adsorption kinetics (5–120 min) in strontium adsorption. Moreover, it also demonstrates impressive distribution coefficients even in real environmental water system. Based on comprehensive analyses using zeta potential, SEM‐EDS, TEM, FT‐IR, XRD, and XPS, it was concluded that the exceptional adsorption performance of Na‐NLMO can be attributed to its accessible layered structure, negatively charged framework, and high affinity for Sr(II). This study has effectively addressed the critical problems associated with strontium adsorbent, including harsh synthesis conditions, insufficient stability, and high interference from acidity, alkali as well as alkaline earth metals.

## 4 Experimental Section

### Materials

Manganese carbonate (MnCO_3_, 99.95%), sodium carbonate anhydrous (Na_2_CO_3_, 99.9%), potassium carbonate (K_2_CO_3_, 99%), lithium carbonate (Li_2_CO_3_, 99.5%), strontium chloride hexahydrate (SrCl_2_·6H_2_O, 99.99%), sodium chloride (NaCl, 99.5%), potassium chloride (KCl, 99.5%), anhydrous calcium chloride (CaCl_2_, 96%), magnesium chloride (MgCl_2_, 99.5%) were sourced from Shanghai Macklin Biochemical Technology Co., Ltd. (China). Hydrochloric acid (HCl) and ethanol anhydrous were sourced from Sinopharm chemical reagent Co, Ltd. (China). All solutions utilized in this study were prepared using ultrapure water with an electrical resistance exceeding 18 MΩ·cm. Tap water, river water, and sea water were collected from the University of South China, Xiangjiang River, and Beihai Bay in China, respectively.

### Characterization techniques

The material Na‐NLMO was prepared via a rapid solid‐phase calcination method. The surface morphology and element composition of materials were characterized by SEM (ZEISS Sigma 300, Germany) coupled with EDS. To investigate the micro‐morphology of the material, droplets of the powder dispersed in water were deposited onto a carbon‐coated copper grid and then air‐dried before imaging with the high‐resolution TEM (FEI Tecnai F20, USA) at an accelerating voltage of 200 kV. XRD patterns were recorded on the Bruker D8 Advance X‐ray diffractometer (Cu Kα radiation, λ = 1.5406 Å) at diffraction angles (2θ) from 10 to 80°. XPS was collected with Thermo Scientific K‐Alpha (USA) using a monochromatic Al Kα X‐ray source (1487 eV) at 12 kV. The heat absorption curves and differential scanning calorimetry (DSC) signals were analyzed using a thermal analyzer (NETZSCH STA 449F3, Germany) in an oxygen atmosphere, with the temperature ranging from 30 to 1200 °C at a heating rate of 10 °C min^−1^. The specific surface area of the adsorbent was determined by the Brunauer–Emmett–Teller (BET) equation using an automatic specific surface area analyzer (TriStar II 3020, USA). The pore volume and pore size distribution were obtained using the Barrett–Joiner–Halenda (BJH) model. The FTIR spectra (KBr pellet) was measured on the IR‐Tracer 100 spectrometer (SHIMADZU, Japan). Zeta potentials at varying pH values (1.0, 2.0, 3.0, 4.0, 5.0, 6.0, 7.0, and 8.0) were determined by Zetasizer Nano (ZEN 3600, Malvern, England).

### Adsorption Experiments

The radioactive adsorption experiments were carried out in a special glove box equipped with lead glass. After adsorption, 2 mL of the aqueous phase from the seawater sample was collected and mixed with 2 mL of scintillation solution (Ultima Gold). The activity (in cpm) of the β‐emitter ^90^Sr was measured using a Tri‐Carb 4910TR (Revvity) liquid scintillation counter. In the non‐radioactive adsorption experiments, SrCl_2_·6H_2_O was employed as an analogue for ^90^Sr to investigate the adsorption behavior of Na‐NLMO. The working solutions of Sr^2+^ with different concentrations of HCl were diluted from the mother liquor and adjusted by adding 0.1 M HCl or 0.1 M NaOH solutions, and the initial concentration of the working solution was fixed at 200 mg L^−1^. The phase ratio (m/V) was set to 0.67 g L^−1^. Unless otherwise indicated, the experiments were performed in 50 mL polyethylene tubes at 298 ± 1 K, and each 20 mg of Na‐NLMO was mixed with 30 mL of working solution and shaken at 160 rpm for 120 min. The concentration of metal ions was determined using an atomic absorption spectrophotometer (AAS‐7000, Shimazu, Japan) at a C_2_H_2_ gas and flow rate of 3 and 15 L min^−1^, respectively. Pseudo‐first‐order (PFO) (Equation S1, Supporting Information), pseudo‐second‐order (PSO) (Equation S2, Supporting Information) and intraparticle diffusion (IPD) (Equation S3, Supporting Information) models were employed to investigate the adsorption kinetics. The Langmuir (Equation S4, Supporting Information), Freundlich (Equation S5, Supporting Information), and Dubinin–Radushkevich (D‐R) (Equations S6 and S7, Supporting Information) models were utilized to elucidate the adsorption mechanism and assess the adsorption capacity of Sr(II). The adsorption capacity (*Q*, mg/g), the removal efficiency (*R*), the desorption efficiency (*D*), and the distribution coefficient *K*_d_ (mL/g) were defined as follows:

(1) $$Q_{e}=\frac{V\cdot \left(C_{i}-C_{e}\right)}{m}$$

(2) $$R\left(\%\right)=\frac{C_{i}-C_{e}}{C_{i}}\times 100\%$$

(3) $$D\left(\%\right)=\frac{C_{e}\cdot V}{m\cdot Q_{e}}\times 100\%$$

(4) $$K_{d}=\frac{V\cdot \left(C_{i}-C_{e}\right)\cdot 1000}{m\cdot C_{e}}$$

where C_i_ and C_e_ represent the initial and equilibrium concentrations of metal ion in the solution, respectively. V is the volume of the solution, while m denotes the mass of the adsorbent.

## Conflict of Interest

The authors declare no conflict of interest.

## Supporting information



Supporting Information

## Data Availability

The data that support the findings of this study are available from the corresponding author upon reasonable request.
