# Supplementary material for: Facile Synthesis of Nanolayered Manganese Oxide for the Efficient and Selective Removal of Strontium(II) from Nuclear Wastewater
Source: Adv Sci (Weinh). 2025 Jun 19;12(30):e17776. doi: 10.1002/advs.202417776 (PMC12376596; doi:10.1002/advs.202417776)
Supplement: Supplementary file 1 — Supporting Information [file ADVS-12-e17776-s001.docx]

*Supporting Information*

**Facile Synthesis of Nanolayered Manganese Oxide for the Efficient and Selective** **Removal of Strontium(II) from Nuclear Wastewater**

Fan Wang ^a, #^, Qi Zheng ^a, #^, Wenya Tai ^b,^ *, Qiang Wu ^a^, Xinglei Li ^a^, Yehuizi Wu ^a^, Ningchao Zheng ^a^, Shunyan Ning ^a^, Deqian Zeng ^a^, Hiroshi Watabe ^c^, Yan Wu ^d^, Hai Li ^e^, Yuezhou Wei ^a, d^ and Xiangbiao Yin ^a, f,^ *

*a School of Nuclear Science and Technology, University of South China, 28 Changsheng West Road, Hengyang 421001, China.*

*b Institute of Zhejiang University - Quzhou, 99 Zheda Road, Quzhou 324000, China*

*c Division of Radiation Protection and Safety Control, Cyclotron and Radioisotope Center, Tohoku University, 6-3 Aoba, Aramaki, Aoba-ku, Sendai,*

*Miyagi 980-8578, Japan.*

*d School of Nuclear Science and Engineering, Shanghai Jiao Tong University, 800 Dong Chuan Road, Shanghai 200240, China.*

*e Hangzhou Xiangting Technology Co., Ltd., 1378 Wenyi West Road, Hangzhou 314000 ,China.*

*f Key Laboratory of Advanced Nuclear Energy Design and Safety, Ministry of Education, University of South China, 28 Changsheng West Road, Hengyang, China.*

# These authors contributed equally to the work and should be regarded as co-first authors.

* To whom correspondence should be addressed.

**1.XRD Patterns of Na-NLMO prepared with different sintering times**

**2. Batch Adsorption Experiments**

**3. Equations**

**4. Effect of adsorbent dose**

**5. Effect of acidity**

**6. Adsorption kinetics**

**7. Adsorption isotherms**

**8. Adsorption mechanism**

**9. Effect of coexisting ions and real wastewater on the adsorption performance of Na-NLMO for Sr^2+^**

**10. Desorption and recycling performance of Na-NLMO for Sr^2+^**

**11. Chemical stability**

**12. The potential of the material**

1. **XRD Patterns of Na-NLMO prepared with different sintering times**


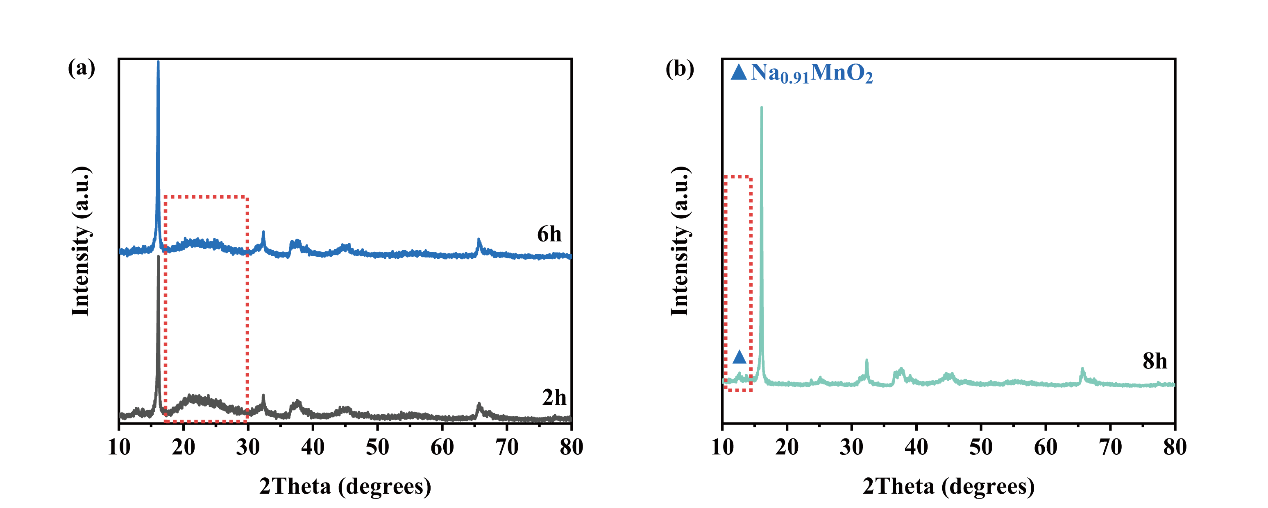


**Fig. S1.** (a) XRD patterns of Na-NLMO calcined for 2 and 6 h. (b) XRD patterns of Na-NLMO calcined for 8 h.

**2. Batch Adsorption Experiments**

In pH- effect experiments of Sr^2+^ adsorption, the pH values of various Sr^2+^ aqueous solutions were adjusted in the pH range from 1 to 8 or from 1 mol/L to 3 mol/L HCl by using 0.1 mol/L HCl and NaOH solutions. The initial Sr^2+^ concentrations were from 196.72 mg/L to 203.06 mg/L. Adsorption experiments of all samples were performed with *V/m* of 30 mL/0.02g, at room temperature and for 2 h contact time. The solid product remaining after the adsorption process was separated from the solution by a 0.45 μm aqueous filter head. Then the concentration of Sr^2+^ in aqueous solutions of different pH values was determined by atomic absorption spectrophotometer (AAS).

The kinetic experiments of Na-NLMO for the Sr^2+^ removal were performed. Kinetic experiments were performed by adding 20 mg of Na-NLMO powder sample to 30 mL solution with an initial Sr^2+^ ion concentration of about 217.10 mg/L at room temperature. The solution pH was adjusted to 3 with 0.1 mol/L HCl. The mixture was placed in a water bath shaker and an appropriate amount of the supernatant was extracted and filtered after the indicated contact time (1, 3, 5, 10, 15, 20, 30, 40, 60, 120 and 240 min). Then the Sr^2+^ concentrations in aqueous solutions at different contact times were determined by AAS.

In isothermal adsorption experiments, HCl solutions at pH=3 with initial Sr^2+^ ion concentrations of 18.14-3084.93 mg/L were prepared separately. The *V/m* ratio of all samples was 30 mL/0.02g. The adsorption lasted for about 2 h contact time at room temperature. The concentration of Sr^2+^ in the supernatant after filtration separation was then tested by AAS.

Competitive ion adsorption studies were carried out (V/m = 30 mL/0.02g) at room temperature, 2-hour contact time. The Sr^2+^ removal experiments of Na-NLMO were tested in the presence of K^+^, Na^+^, Ca^2+^, Mg^2+^, Sr^2+^. The Sr^2+^ removal experiments of Na-NLMO in actual water samples were conducted by adding Sr^2+^ ions to tap water (Hengyang, Hunan, China), river water (Hengyang, Hunan, China) and sea water (Beihai, Guangxi, China) to simulate water bodies contaminated with Sr^2+^ ions.

**3. Equations**

The pseudo-first, pseudo-second order kinetic ^[1]^ and intra-particle diffusion models ^[2]^ are shown in **Eq. S1**, **S2** and **S3**.

$\ln\left(Q_{e}-Q_{t}\right)=\ln Q_{e}-k_{1}t$ (Eq. S1)

$\frac{t}{Q_{t}}=\frac{1}{k_{2}Q_{e}^{2}}+\frac{t}{Q_{e}}$ (Eq. S2)

$Q_{t}=k_{id}t^{1/2}+C_{\text{i}}$ (Eq. S3)

where Q_t_ and Q_e_ (mg/g) are the amounts of Sr^2+^ absorbed at time t (min) and at equilibrium time, respectively, while *k*_1_ (min^-1^) and *k*_2_ (g mg^-1^ min^-1^) are pseudo-first-order and the pseudo-second-order rate constant of kinetics models, respectively. *k*_1_ reflects the rate of change in adsorption capacity over time, with a larger value indicating a faster adsorption rate; *k*_2_ (g mg^-1^ min^-1^) measures the number of active sites on the surface, and a higher value suggests a faster adsorption rate.

The intra-particle diffusion model (**Eq. S3**) was employed to investigate the adsorption mechanism and the rate-limiting steps of the adsorption process. *k*_id_ (mg/(g min^1/2^)) is the diffusion constant, and C_i_ is the thickness of the liquid film.

The isotherm adsorption models of Langmuir (**Eq. S4**), Freundlich (**Eq. S5**) ^[3]^, and Dubinin-Radushkevich (D-R) (**Eq. S6** and **S7**) ^[4]^ were employed to analyze the experimental data for Sr(II), respectively.

$Q_{e}=\frac{Q_{\text{m}}K_{L}C_{e}}{1+K_{L}C_{e}}$ (Eq. S4)

$Q_{\text{e}}=K_{F}C_{e}^{1/n}$ (Eq. S5)

$\text{ln}Q_{e}=\ln Q_{\max}-\beta e^{2}$, $e=RT\ln\left(1+\frac{1}{C_{e}}\right)$ (Eq. S6)

$E=\frac{1}{\sqrt{2\beta}}$ (Eq. S7)

Q_max_ (mg/g) is the maximal equilibrium adsorption capacity, *K*_L_ (L/mg) is the Langmuir model constant, and *K*_F_ ((mg/g)/(mg/L)^1/n^) and n are the Freundlich model adsorption equilibrium constants. *β* (mol^2^/J^2^) is the adsorption energy constant, *e* (kJ/mol) is the Polanyi potential energy, *R* is the general gas constant (8.314 J.mol^-1^.K^-1^), and *T* is the absolute temperature. *E* (kJ/mol) shows the adsorption free energy.

**4. Effect of solid-liquid ratio**


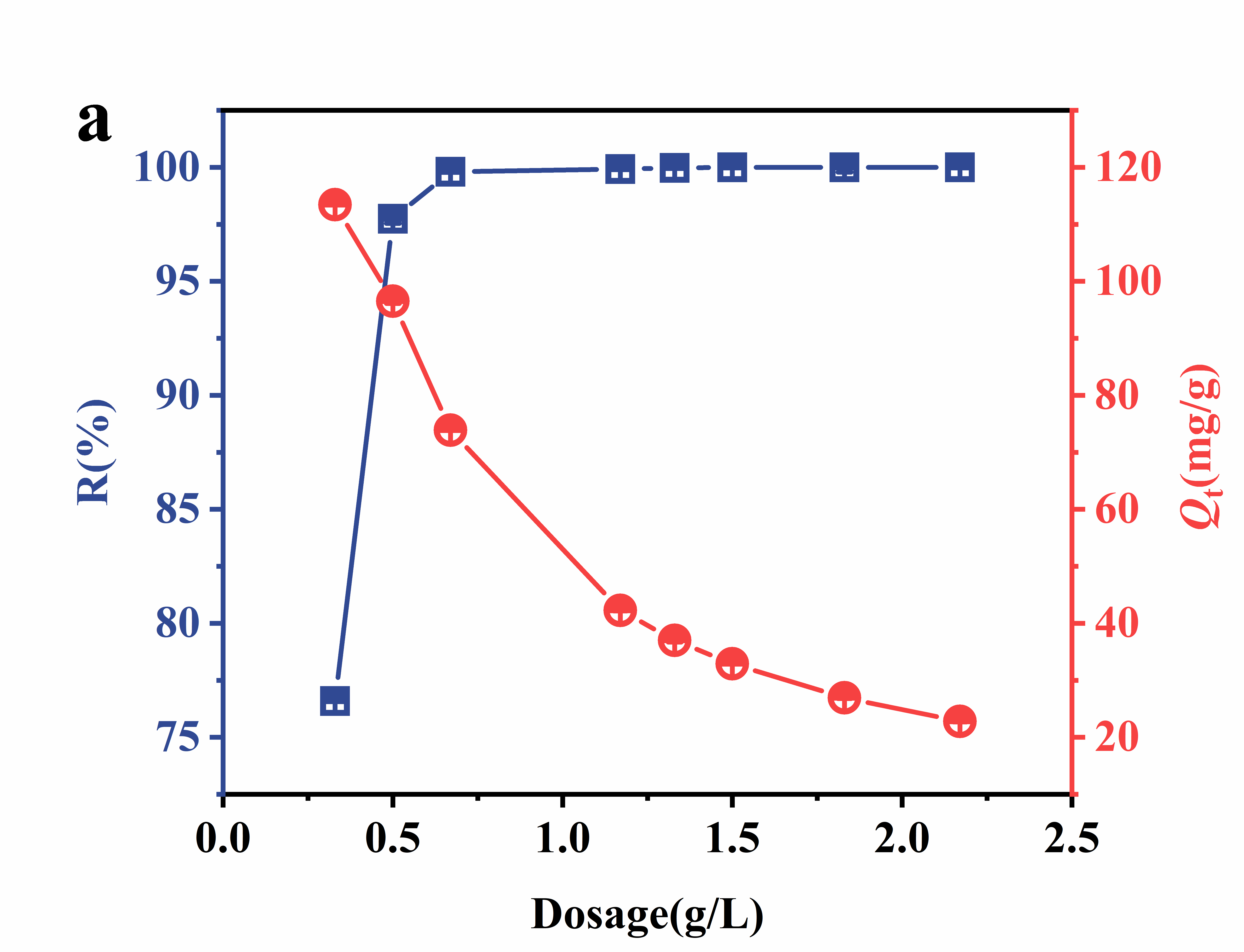


**Fig. S2.** Influence of the Na-NLMO dosage on Sr^2+^ removal. T = 25℃, pH=3, [Sr(II)] = 50 mg/L, t = 2 h.

**5. Effect of acidity**

**Table S1.** The pH- effect adsorption results of Na-NLMO for Sr^2+^ removal.

| pH or *C* _HCl_ | *C*_i_ (mg/L) | *C*_e_ (mg/L) | *Q*_e_(mg/g) |
| --- | --- | --- | --- |
| 8 | 198.62 | 138.52 | 90.15 |
| 7 | 197.39 | 137.82 | 89.35 |
| 6 | 199.72 | 140.37 | 89.02 |
| 5 | 203.06 | 142.11 | 91.43 |
| 4 | 196.72 | 135.21 | 92.27 |
| 3 | 201.39 | 122.62 | 118.16 |
| 2 | 201.73 | 136.12 | 98.42 |
| 1 | 202.40 | 152.87 | 74.30 |
| 1 M HCl | 199.51 | 179.79 | 29.58 |
| 2 M HCl | 201.92 | 186.49 | 23.15 |
| 3 M HCl | 200.64 | 189.25 | 17.08 |

**6. Adsorption kinetics**

**Table S2.** The Sr^2+^ adsorption kinetics results of Na-NLMO.

| *t* (min) | *C*_t_ (mg/L) | *Q*_t_(mg/g) |
| --- | --- | --- |
| 0 | 217.10 | 0.00 |
| 1 | 184.83 | 48.41 |
| 3 | 179.87 | 55.84 |
| 5 | 174.13 | 64.45 |
| 10 | 163.81 | 79.94 |
| 15 | 158.45 | 87.98 |
| 20 | 153.48 | 95.43 |
| 30 | 149.77 | 101.00 |
| 40 | 146.53 | 105.85 |
| 60 | 143.78 | 109.98 |
| 120 | 141.16 | 113.91 |
| 240 | 141.15 | 113.93 |

**7. Adsorption isotherms**

**Table S3.** Experimental results of the adsorption capacity study of Na-NLMO in strontium-containing solutions.

| *C*_i_ (mg/L) | *C*_e_ (mg/L) | *Q*_e_(mg/g) |
| --- | --- | --- |
| 18.14 | 0.00 | 27.21 |
| 38.55 | 0.00 | 57.83 |
| 61.35 | 9.78 | 77.36 |
| 82.18 | 26.91 | 82.91 |
| 104.59 | 31.12 | 110.21 |
| 215.06 | 130.59 | 126.70 |
| 428.52 | 337.23 | 136.93 |
| 831.55 | 736.31 | 142.86 |
| 1051.70 | 955.83 | 143.81 |
| 2079.70 | 1983.57 | 144.19 |
| 3084.93 | 2988.58 | 144.52 |

**8. Adsorption mechanism**

Fig. S3 illustrates the XPS patterns of the Mn *3s* before and after strontium adsorption by Na-NLMO. The results show that there are two splitting peaks of Mn and the amplitude of the splitting peaks is near 4.7 eV ^[5]^, indicating that Mn mainly exists as Mn^4+^ in the material. The binding energy is not shifted greatly after adsorption, which proves that the adsorption process is mainly electrostatic interaction and ion exchange, which is consistent with the conclusion drawn in the paper.


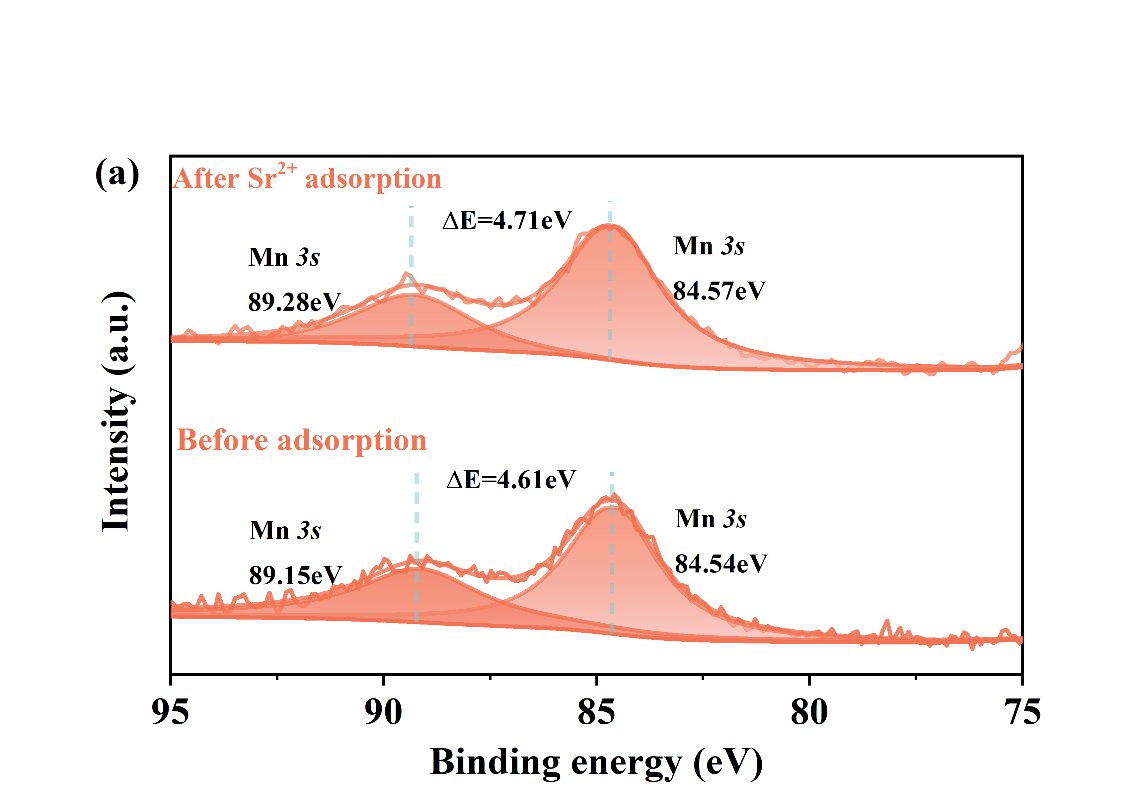


**Fig. S3.** Mn *3s* XPS spectra of the Na-NLMO before and after Sr^2+^ adsorption

**9. Effect of coexisting ions and real wastewater on the adsorption performance of Na-NLMO for Sr^2+^**

**Table S4.** Adsorption results of Na-NLMO under different Na/Sr molar ratios.

| Molar Ratios (Na/Sr) | Sr^2+^ | *C*_0_ (mg/L) | ±*C*_0_  (mg/L) | *C*_e_ (mg/L) | ±*C*_e_  (mg/L) | | *K*_d_  (mL/g) | ±*K*_d_ (mL/g) |
| --- | --- | --- | --- | --- | --- | --- | --- | --- |
| 1 |  | 5.05 | 0.0515 | 0.067 | 0.0055 | 112360.6 | | 9346.76 |
| 10 |  | 5.10 | 0.1010 | 0.081 | 0.0030 | 93092.7 | | 3503.43 |
| 100 |  | 5.37 | 0.3725 | 0.100 | 0.0010 | 79500.6 | | 814.08 |
| 1000 |  | 5.67 | 0.6650 | 0.104 | 0.0053 | 80218.0 | | 4115.30 |

**Table S5.** Adsorption results of Na-NLMO under different K/Sr molar ratios.

| Molar Ratios (K/Sr) | Sr^2+^ | *C*_0_ (mg/L) | ±*C*_0_  (mg/L) | *C*_e_ (mg/L) | ±*C*_e_  (mg/L) | | *K*_d_  (mL/g) | ±*K*_d_ (mL/g) |
| --- | --- | --- | --- | --- | --- | --- | --- | --- |
| 1 |  | 4.98 | 0.0245 | 0.037 | 0.0010 | 200356.91 | | 5455.59 |
| 10 |  | 5.37 | 0.3725 | 0.053 | 0.0010 | 164064.41 | | 2764.58 |
| 100 |  | 5.77 | 0.7690 | 0.054 | 0.0035 | 147787.31 | | 11037.63 |
| 1000 |  | 5.21 | 0.2055 | 0.060 | 0.0005 | 129740.36 | | 1102.86 |

**Table S6.** Adsorption results of Na-NLMO under different Mg/Sr molar ratios.

| Molar Ratios (Mg/Sr) | Sr^2+^ | *C*_0_ (mg/L) | ±*C*_0_  (mg/L) | *C*_e_ (mg/L) | ±*C*_e_  (mg/L) | | *K*_d_  (mL/g) | ±*K*_d_ (mL/g) |
| --- | --- | --- | --- | --- | --- | --- | --- | --- |
| 1 |  | 5.06 | 0.0600 | 0.027 | 0.0005 | 284960.47 | | 5404.91 |
| 10 |  | 5.20 | 0.2000 | 0.034 | 0.0015 | 231983.04 | | 10454.46 |
| 100 |  | 5.21 | 0.2100 | 0.034 | 0.0003 | 229868.25 | | 1713.84 |
| 1000 |  | 5.16 | 0.1600 | 0.040 | 0.0003 | 193356.76 | | 1225.51 |

**Table S7.** Adsorption results of Na-NLMO under different Ca/Sr molar ratios.

| Molar Ratios (Ca/Sr) | Sr^2+^ | *C*_0_ (mg/L) | ±*C*_0_  (mg/L) | *C*_e_ (mg/L) | ±*C*_e_  (mg/L) | | *K*_d_  (mL/g) | ±*K*_d_ (mL/g) |
| --- | --- | --- | --- | --- | --- | --- | --- | --- |
| 1 |  | 5.52 | 0.5200 | 0.067 | 0.0005 | 122055.39 | | 922.06 |
| 10 |  | 5.12 | 0.1200 | 0.089 | 0.0005 | 84828.57 | | 484.99 |
| 100 |  | 5.25 | 0.2500 | 2.255 | 0.0058 | 1990.65 | | 8.90 |
| 1000 |  | 7.23 | 2.2300 | 6.227 | 0.0225 | 241.87 | | 6.29 |

**Table S8.** Selective Sr^2+^ capture ability under the coexistence of K^+^/Ca^2+^/Na^+^/Mg^2+^/Sr^2+^.

| Ions | *C*_0_  (mg/L) | ±*C*_0_  (mg/L) | *C*_e_ (mg/L) | ±*C*_e_  (mg/L) | *K*_d_  (mL/g) | ±*K*_d_  (mL/g) | *R*  (%) | ±*R*  (%) |
| --- | --- | --- | --- | --- | --- | --- | --- | --- |
| K^+^ | 4.86 | 0.1400 | 0.495 | 0.0168 | 13260.73 | 499.73 | 89.83 | 0.34 |
| Ca^2+^ | 4.74 | 0.2600 | 0.221 | 0.0153 | 30890.14 | 74.8162 | 95.35 | 0.32 |
| Na^+^ | 5.07 | 0.0700 | 0.511 | 0.0213 | 12054.29 | 428.39 | 81.08 | 0.29 |
| Mg^2+^ | 4.95 | 0.0500 | 0.284 | 0.0220 | 24812.83 | 2038.32 | 94.26 | 0.44 |
| Sr^2+^ | 4.93 | 0.0700 | 0.028 | 0.0015 | 268047.41 | 14702.59 | 99.44 | 0.03 |

**Table S9.** The results on Sr^2+^ ions removal by Na-NLMO in actual water samples contaminated with Sr^2+^ ions.

| Samples | *C*_0_  (mg/L) | | ±*C*_0_  (mg/L) | *C*_e_ (mg/L) | ±*C*_e_ (mg/L) | *K*_d_  (mL/g) | ±*K*_d_ (mL/g) |
| --- | --- | --- | --- | --- | --- | --- | --- |
| Sea water | | 10.61 | 5.6100 | 5.470 | 0.0900 | 1410.02 | 47.88 |
| Lake water | | 5.25 | 0.2500 | 0.014 | 0.0010 | 563400 | 40350 |
| Tap Water | | 5.18 | 0.1800 | 0.015 | 0.0010 | 518912.95 | 34694.20 |

**10. Desorption and recycling performance of Na-NLMO for Sr^2+^**


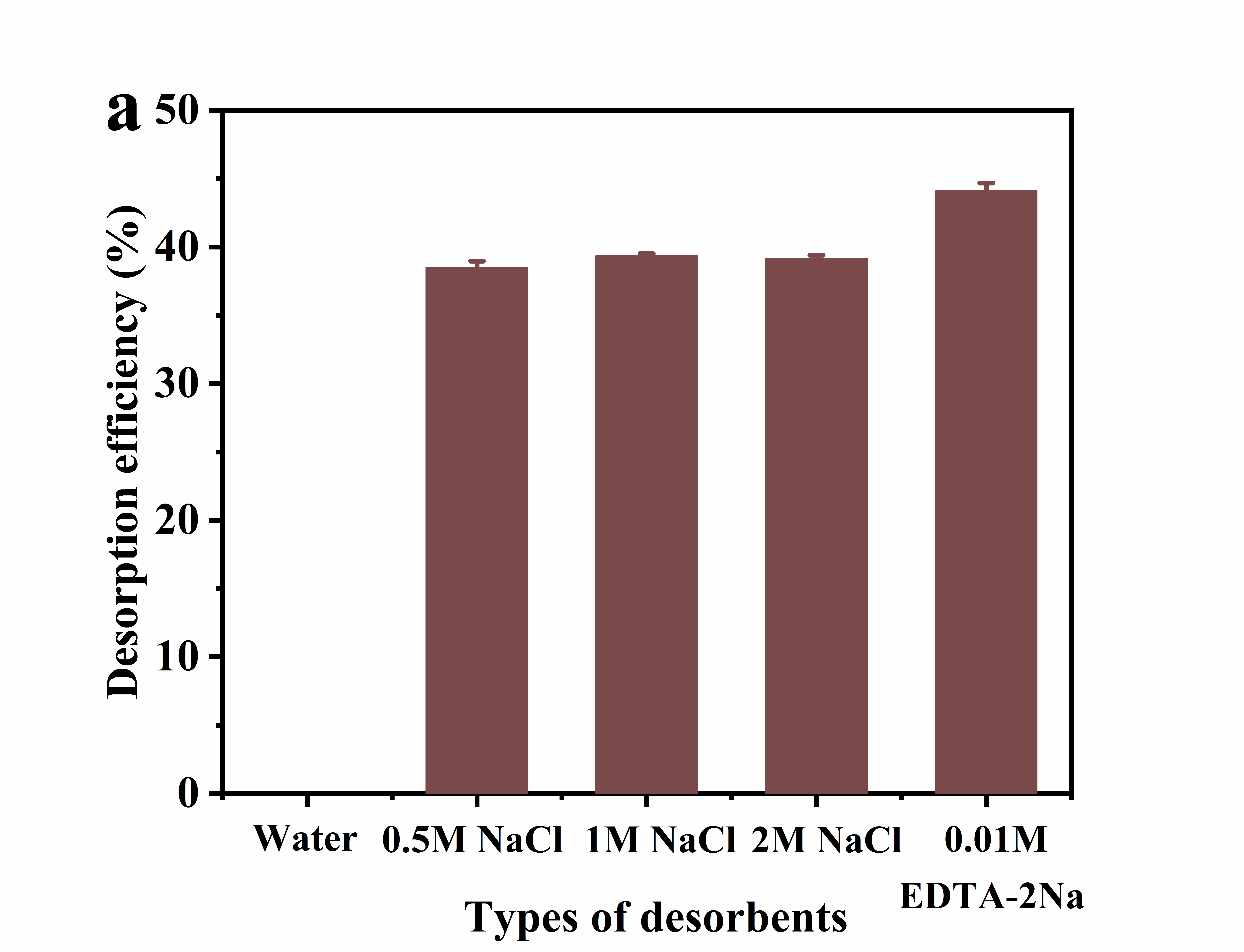


**Fig. S4. Effect of different desorbent on desorption efficiency.**

**Table S10.** Desorption efficiency of different desorbent types for Sr^2+^ in Na-NLMO

| Desorption agent | *C*_0_  (mg/L) | | ±*C*_0_  (mg/L) | *C*_e_ (mg/L) | ±*C*_e_ (mg/L) | *D*  (%) | ±*D* (%) |
| --- | --- | --- | --- | --- | --- | --- | --- |
| Water | | 10.228 | 0.2400 | 0 | 0 | 0 | 0 |
| 0.5M HCl | | 10.228 | 0.2400 | 7.750 | 0.0200 | 75.77 | 0.24 |
| 1M HCl | | 10.228 | 0.2400 | 10.214 | 0.1425 | 99.82 | 0.56 |
| 0.5M NaCl | | 10.228 | 0.2400 | 3.942 | 0.2501 | 38.52 | 0.44 |

**Table S11.** Desorption efficiency of Sr^2+^ at different desorption times under 1M HCl conditions

| Time (h) | *C*_0_  (mg/L) | | ±*C*_0_  (mg/L) | *C*_e_ (mg/L) | ±*C*_e_ (mg/L) | *D*  (%) | ±*D* (%) |
| --- | --- | --- | --- | --- | --- | --- | --- |
| 2 | | 10.228 | 0.2400 | 8.206 | 0.2734 | 80.27 | 0.48 |
| 4 | | 10.228 | 0.2400 | 10.200 | 0.5800 | 99.73 | 0.35 |
| 24 | | 10.228 | 0.2400 | 10.214 | 0.1425 | 99.82 | 0.56 |

**Table S12.** Recycling performance of Na-NLMO on Sr^2+^

| Cycle number | *C*_0_  (mg/L) | | ±*C*_0_  (mg/L) | *C*_e_ (mg/L) | ±*C*_e_ (mg/L) | *R*  (%) | ±*R* (%) |
| --- | --- | --- | --- | --- | --- | --- | --- |
| 1 | | 10.228 | 0 | 0 | 0 | 100 | 0.16 |
| 2 | | 10.046 | 0.0400 | 0.002 | 0.1500 | 99.98 | 0.32 |
| 3 | | 10.104 | 0.1501 | 0.004 | 0.2213 | 99.96 | 0.59 |
| 4 | | 10.125 | 0.0802 | 0.006 | 0.2605 | 99.94 | 0.27 |

**11. Chemical stability**


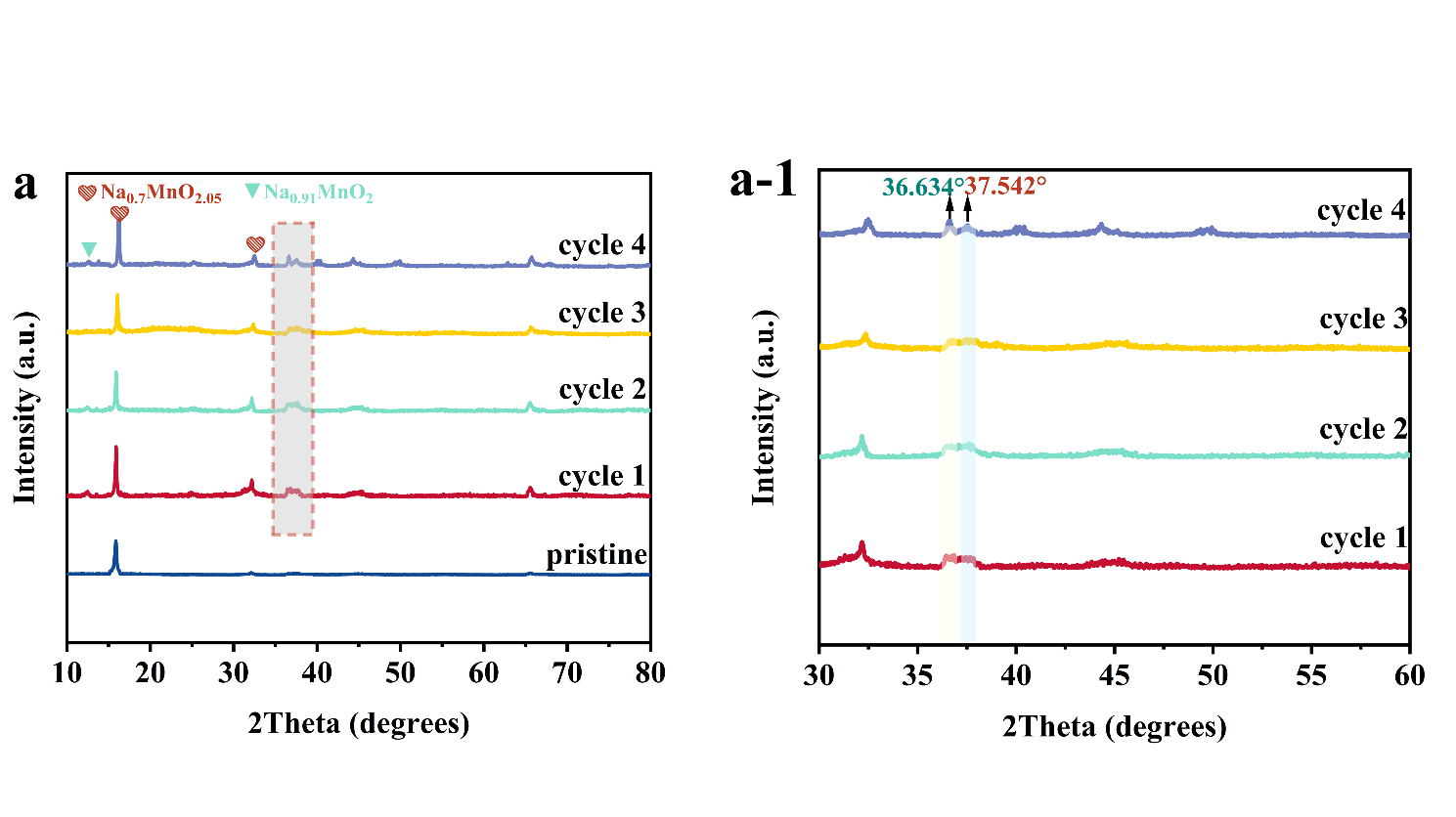


**Fig. S5. (a)** XRD patterns and (a-1) enlarged patterns of Na-NLMO after adsorption-desorption cycle.


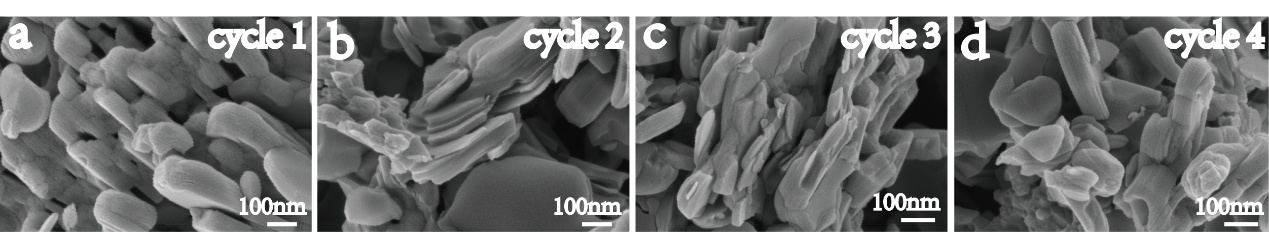


**Fig. S6.** (a)-(d) SEM images of Na-NLMO after adsorption-desorption cycle.


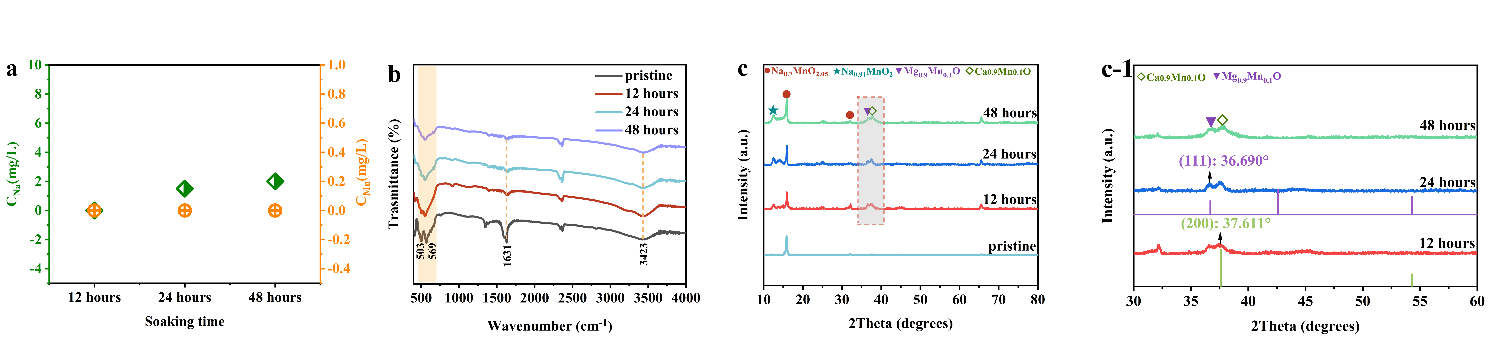


**Fig. S7.** (a) Effect of soaking time in seawater on the dissolution behavior of Na and Mn in Na-NLMO; (b) FT-IR spectra, (c) XRD patterns and (c-1) enlarged patterns of Na-NLMO after immersion in seawater for different durations.


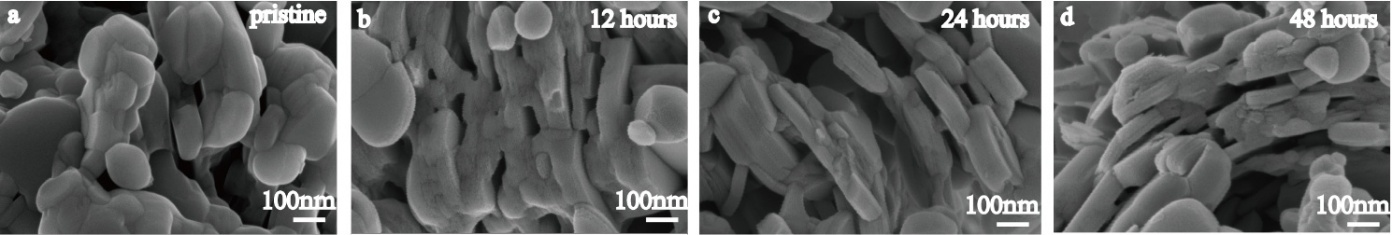


**Fig. S8.** (a)-(d) SEM images of Na-NLMO after immersion in seawater for different durations.


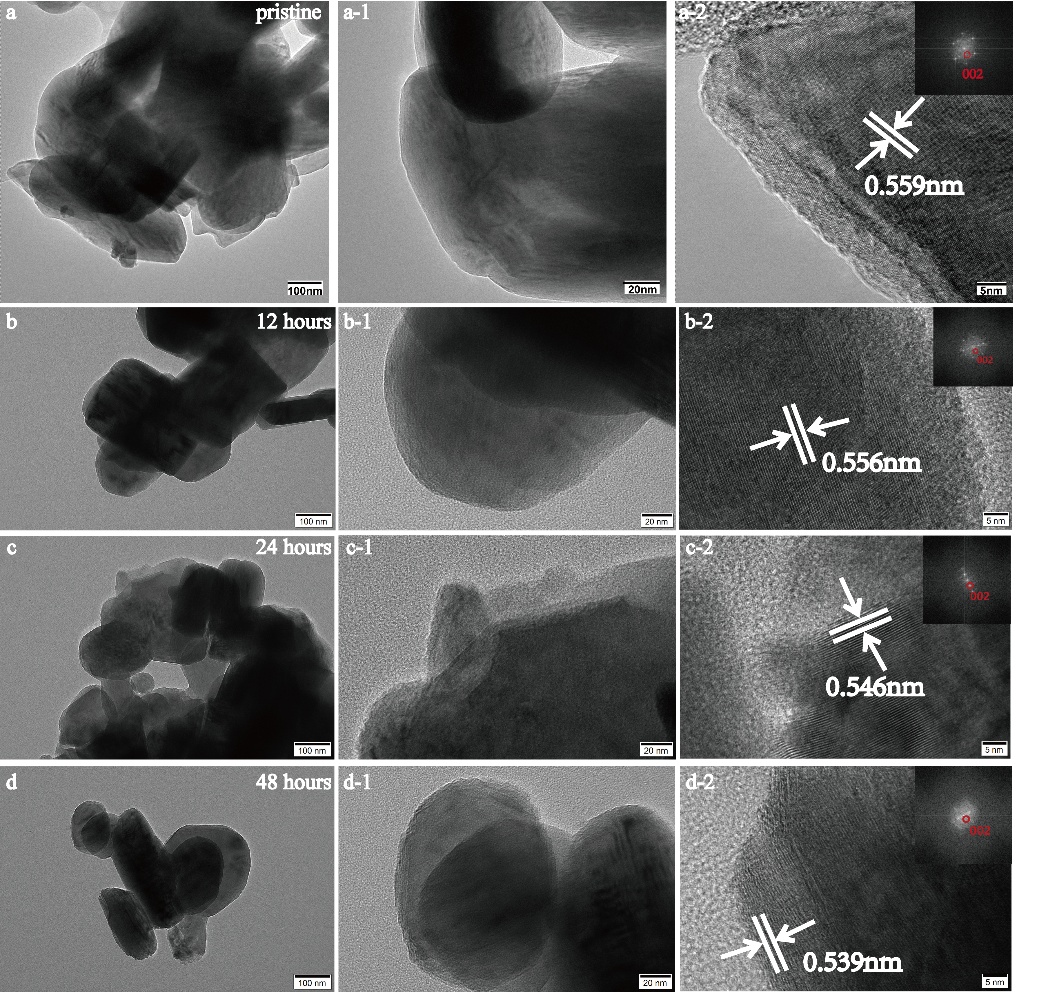


**Fig. S9.** (a)-(d) TEM images of Na-NLMO after immersion in seawater for different durations.

**12. The potential of the material**

The Na-NLMO ion exchanger was synthesised through a straightforward solid-phase reaction, utilizing MnCO₃ and Na₂CO₃ in conjunction with anhydrous ethanol. This simple, time-efficient method offers several advantages in terms of scalability and reproducibility, which are critical for its potential industrial application. In this section, we discuss the broader implications of this work, focusing on scalability, reactor design optimization, and strategies to bridge the gap between laboratory-scale development and industrial implementation.

The solid-phase synthesis method employed in this study can be easily adapted for large-scale production. The grinding procedure, which ensures a homogeneous dispersion of reactants, can be scaled by replacing manual grinding with mechanized ball mills or continuous grinding systems. These alternatives could significantly increase throughput while maintaining the uniformity of the product. Additionally, the moderate reaction temperature and short processing time (e.g., calcination at 500 ℃ for 4 hours) make this process energy-efficient, further enhancing its appeal for industrial-scale operations. As such, we anticipate that integration with automated material handling and large-scale calcination furnaces could yield high quantities of Na-NLMO material with consistent quality.

In terms of reactor design, the use of a traditional muffle furnace in this study is suitable for laboratory-scale synthesis but may present limitations in terms of scalability and heat transfer efficiency when transitioning to industrial-scale production. We propose that continuous or rotary kilns, which allow for uniform temperature control and longer residence times, could be explored for larger-scale calcination processes. The optimal reactor design would need to balance efficient heat distribution with material flow to prevent thermal degradation or uneven phase formation. Furthermore, optimizing the heat exchange of reactor and material handling systems would be key to improving energy efficiency and minimizing product loss during scale-up.

One of the key challenges in scaling the Na-NLMO synthesis process is ensuring that the material retains its desired ion-exchange capacity and purity under real-world conditions, such as variable feed compositions in industrial applications. In our current laboratory setting, the ion-exchange performance of Na-NLMO is stable and effective for Sr²⁺ removal. However, translating these results to large-scale industrial processes, particularly for water treatment or ion-exchange applications, would require additional considerations. Potential issues such as particle agglomeration, reduced washing efficiency, and loss of active sites during handling must be addressed. Automation of washing and drying stages using continuous filtration systems or advanced drying techniques could help mitigate these challenges. Additionally, real-time monitoring and quality control techniques, such as in-line spectroscopy or X-ray diffraction, could be employed to ensure consistent product quality during large-scale synthesis.

Further research should focus on recycling Na-NLMO materials in real-world environments for extended periods of time to evaluate its long-term stability. Additionally, optimization of the synthesis method for higher material throughput and improved cost-efficiency will be crucial in facilitating its transition from laboratory-scale development to full-scale industrial application. We also recommend investigating alternative, greener synthesis routes that minimize energy consumption and waste production, which would align the production process with sustainable manufacturing practices.

**References**

[1] Li, C.; Chen, D.; Ding, J.; Shi, Z. A novel hetero-exopolysaccharide for the adsorption of methylene blue from aqueous solutions: Isotherm, kinetic, and mechanism studies. J. Clean. Prod. 2020, 265, 121800, DOI 10.1016/j.jclepro.2020.121800.

[2] Pan, S.; Zhang, X.; Qian, J.; Lu, Z.; Hua, M.; Cheng, C.; Pan, B. A new strategy to address the challenges of nanoparticles in practical water treatment: mesoporous nanocomposite beads via flash freezing. Nanoscale. 2017, 9, 19154-19161, DOI 10.1039/C7NR06980D.

[3] Wu, J.; Xu Z,; Zhang, W.; Lv, L.; Pan, B.; Nie, G.; Li, M.; Du, Q. Application of heterogeneous adsorbents in removal of dimethyl phthalate: Equilibrium and heat. Aiche. J. 2010, 56, 2699-2705, DOI 10.1002/aic.12175.

[4] Mishra, P.K.; Kumar, R.; Rai, P. K. Surfactant-free one-pot synthesis of CeO_2_, TiO_2_ and Ti@Ce oxide nanoparticles for the ultrafast removal of Cr (VI) from aqueous media. Nanoscale. 2018, 10, 7257-7269, DOI 10.1039/c7nr09563e.

[5] Mark C. Biesinger, Brad P. Payne, Andrew P. Grosvenor, Leo W.M. Lau, Andrea R. Gerson, Roger St. C. Smart, Resolving surface chemical states in XPS analysis of first row transition metals, oxides and hydroxides: Cr, Mn, Fe, Co and Ni, Applied Surface Science, 2011，7，2717-2730，DOI 10.1016/j.apsusc.2010.10.051.
